# Tool Temperature Analysis in Face Milling Using an ESP32-C3-Based Measurement Approach

**DOI:** 10.3390/s26216839

**Published:** 2026-10-29

**Authors:** Hui Liu, Alexander Tillmann, Markus Meurer, Thomas Bergs

**Affiliations:** 1 Manufacturing Technology Institute (MTI), RWTH Aachen University, Campus-Boulevard 30, 52074 Aachen, Germany; a.tillmann@mti.rwth-aachen.de (A.T.); m.meurer@mti.rwth-aachen.de (M.M.); t.bergs@mti.rwth-aachen.de (T.B.); 2 Fraunhofer Institute for Production Technology IPT, Steinbachstr. 17, 52074 Aachen, Germany

**Keywords:** face milling, tool temperature, thermocouple, ESP32-C3-based system, data acquisition, finite element modeling, heat partitioning, flood cooling

## Abstract

Reliable temperature measurement on rotating milling tools remains challenging, particularly under flood cooling, where optical access and sensor integration are restricted. This study develops a compact, tool-integrated measurement system combining an embedded Type-K thermocouple, analog signal conditioning, ESP32-C3-based data acquisition, local microSD storage, and Wi-Fi data transfer. A sealed housing enables operation under cutting-fluid exposure, while local storage decouples acquisition from wireless communication. The system was evaluated through face-milling experiments on 42CrMo4 steel at three cutting speeds and three feed-per-tooth levels under dry cutting and flood cooling. Within the investigated conditions, feed per tooth exerted the strongest influence on the measured internal tool temperature during dry cutting. Temperatures reached approximately 175–235 °C under dry cutting and remained generally below 70 °C under flood cooling, which also reduced their sensitivity to cutting parameters. The measurements additionally supported inverse calibration of a three-dimensional finite element model to investigate heat transfer and heat partitioning. From an industrial perspective, the system provides an accessible approach to acquiring internal tool-temperature data during dry and flood-cooled milling without specialized rotary telemetry hardware. These measurements can support cooling-strategy assessment and thermally informed process-parameter selection, while providing a foundation for future tool-condition monitoring and adaptive machining.

## 1. Introduction

During metal cutting, approximately 90% of the mechanical energy supplied to the cutting zone is converted into heat through plastic deformation of the workpiece material and friction at the tool–chip and tool–workpiece interfaces [1]. The resulting temperature rise can accelerate thermally activated wear mechanisms, including diffusion, oxidation, and adhesion, thereby reducing tool life and increasing production costs [2]. Elevated temperatures may also promote coating degradation and adversely affect dimensional accuracy, surface integrity, and process stability. Understanding the thermal behavior of cutting tools is therefore essential for selecting appropriate cutting parameters, assessing cooling strategies, and developing thermally robust machining processes.

Temperature measurement in milling is particularly challenging because of the intermittent nature of tool engagement. Each cutting edge periodically enters and exits the workpiece, resulting in cyclic heating during material removal and cooling during the non-cutting phase of each revolution. These repeated thermal cycles produce pronounced transient temperature variations and may promote thermal fatigue [2]. In addition, cutter rotation, restricted access to the tool–chip contact zone, obstruction by chips, interference from cutting fluid, and short engagement times complicate the acquisition of representative temperature signals under machining conditions.

Various experimental methods for measuring cutting temperatures have been reviewed in the literature [3,4]. Radiation-based techniques, particularly infrared thermography and pyrometry, are widely used because they provide non-contact measurements with high temporal resolution and do not mechanically interfere with the cutting process. Infrared thermography is especially suitable for observing accessible surfaces of the tool, chip, and workpiece. Saleem et al. [5] applied thermal imaging to face milling of Inconel 625 and investigated the relationships among cutting parameters, temperature, and tool wear. Moreira et al. [6] used a high-speed infrared camera to analyze temperature variations under twelve dry face-milling conditions for American Iron and Steel Institute (AISI) H13 steel. Their uncertainty analysis identified surface emissivity and camera viewing angle as the dominant contributors to the combined measurement uncertainty. Zagórski et al. [7] demonstrated that high-speed thermography can capture the temperatures of discrete chips during dry rough milling, although the results were influenced by depth of field, focal position, random chip motion, and camera frame rate.

Pyrometry has likewise been used to measure cutting-tool temperatures. Ueda et al. [8] embedded an optical fiber connected to a pyrometer in the workpiece and recorded the tool temperature as the cutting edge passed a predefined observation position. This arrangement reduced the influence of radiation from surrounding surfaces and was particularly suitable for high-speed cutting. Yang et al. [9] adopted a similar approach, observing the tool surface through a small hole in the workpiece. Although this configuration avoids modifying the cutting tool, the available measurement interval is very short, and the observation hole may locally alter the continuity and geometry of tool engagement. Espinoza-Torres et al. [10] used pyrometry in dry face milling and reported increasing cutting-edge temperatures with increasing cutting speed and feed rate.

Despite their rapid response, radiation-based techniques are constrained by the limited optical accessibility of the cutting zone. Chips, the rotating cutter body, cutting fluid, and surrounding machine-tool components may obstruct the field of view. Reliable measurements also require careful consideration of surface emissivity, viewing angle, reflections, detector wavelength range, and the size and location of the observed area. Consequently, these techniques may not accurately capture the temperature at the tool–chip interface and are difficult to apply reliably under flood-cooling conditions.

Contact-based sensors, particularly thermocouples, provide an alternative means of measuring cutting temperatures. Owing to their compact size, robustness, and comparatively low cost, thermocouples can be embedded in small holes close to the cutting edge. Unlike radiation-based techniques, they remain applicable when the cutting zone is not directly visible or when chips and cutting fluid obstruct the optical path. Their implementation in milling is nevertheless challenging because the sensor and its wiring rotate with the cutter. Conventional data-acquisition systems generally rely on stationary cable connections, which can be readily implemented in turning but are unsuitable for direct use with rotating milling tools.

Slip rings offer one solution by transferring thermocouple signals from the rotating cutter to a stationary data-acquisition system. Kesriklioglu et al. [11] fabricated Type-K thin-film thermocouples on the rake face of carbide inserts and transmitted the resulting millivolt-level thermoelectric signals through a slip ring to an external acquisition unit. Similarly, Yin et al. [12] integrated a thin-film thermocouple array on the flank face of a milling insert and used a conductive slip ring to transmit temperature signals from the rotating cutter. Compared with a single-point thermocouple, the array enabled the thermal response to be recorded at multiple locations on the insert. However, slip-ring systems require careful mechanical integration. They must generally be mounted coaxially with the spindle or tool holder while maintaining adequate dynamic balance, mechanical integrity, and secure wiring [11]. The additional components increase the rotating mass, occupy installation space, and may complicate tool changes. Furthermore, contamination of the sliding contacts by chips, oil mist, cutting fluid, or dust can cause corrosion, fluctuations in contact resistance, and signal instability.

Alternatively, temperature signals can be stored locally or transmitted wirelessly from an acquisition unit mounted on the rotating tool. Liu et al. [13] embedded thermocouples within a peripheral milling cutter to measure the tool-temperature distribution under dry cutting conditions and transmitted the acquired signals through a Wi-Fi module integrated into the tool holder. Augspurger et al. [14] installed Type-K thermocouples in a rotating milling insert or cutter body and used a commercial rotary telemetry system to compare the thermal response under minimum-quantity lubrication and cryogenic cooling conditions. These studies demonstrate the potential of tool-integrated wireless systems to avoid sliding electrical contacts and reduce the mechanical integration requirements associated with slip rings. The specialized telemetry hardware and instrumented-tool configurations used in these approaches motivate the investigation of compact acquisition architectures based on standard electronic modules.

Recent advances in low-cost microcontrollers and compact sensor interfaces provide new opportunities for tool-integrated process monitoring. The ESP32-C3 platform combines onboard data processing, wireless communication, compact dimensions, and low hardware cost. When coupled with a suitable thermocouple interface and local power supply, it can form the basis of a self-contained temperature-measurement system mounted on a rotating tool. Its programmable architecture enables adaptation of the sampling strategy, signal processing, wireless transmission, and synchronization with machining parameters. Nevertheless, the suitability of such a system for quantitative temperature analysis cannot be inferred solely from its electronic specifications. Milling imposes demanding operating conditions, including vibration, centrifugal loading, electromagnetic interference, restricted installation space, and rapidly varying thermal signals. In addition, the measured temperature is governed not only by the sensor and acquisition electronics but also by the periodic heat input associated with interrupted cutting. Experimental validation and systematic evaluation under different machining conditions are therefore required.

Against this background, the present study introduces an integrated experimental–numerical approach for investigating tool temperatures and heat partitioning during face milling under dry and flood cooling conditions. The primary technical contribution is the development of a compact, tool-integrated temperature measurement system based on an embedded Type-K thermocouple, an ESP32-C3 microcontroller, and a compact, sealed acquisition unit. By combining local data storage, wireless communication, and battery-powered operation, the system enables temperature acquisition directly on a rotating milling cutter without relying on specialized rotary telemetry hardware. Its measurement performance is evaluated through electrical verification and quantitative comparison with a commercial MANNER telemetry system under dry cutting conditions, while its applicability under flood cooling is demonstrated experimentally. The scientific and methodological contribution lies in coupling the measured internal tool temperatures with a three-dimensional finite element model through inverse thermal calibration. For each cutting condition, the heat-source intensity is identified from the measured end-of-cut temperature, while the transient temperature histories are compared to assess the simulated heating response. The calibrated model is subsequently employed to reconstruct the spatial and temporal temperature distribution and analyze heat partitioning under different cutting parameters and cooling conditions. Building on previous investigations of tool-temperature modeling in turning and side milling [15,16], this study extends the experimental–numerical methodology to face milling through an accessible measurement platform. The resulting thermal predictions are interpreted within the investigated process window, while independent validation of the reconstructed temperature field and assessment of model transferability remain subjects for future research.

The remainder of this paper is organized as follows: Section 2 describes the development of the integrated temperature measurement system and the experimental setup. Section 3 presents the finite element thermal model and its boundary conditions. Section 4 discusses the experimental and numerical results, focusing on measurement performance, transient tool-temperature behavior, and the influence of cutting parameters and cooling conditions on thermal loading. Finally, Section 5 summarizes the main findings and outlines directions for future research.

## 2. Experimental Setup and Development of the Temperature Measurement System

This section presents the development of the temperature measurement system and the experimental methodology used to evaluate its performance under representative cutting conditions. First, the design and implementation of the temperature-measuring tool holder are described, with particular emphasis on sensor integration and the requirements associated with measurements on a rotating milling tool. The experimental setup for the cutting tests is then introduced, including the machine tool, cutting tool, workpiece material, measurement equipment, cutting parameters, and cooling conditions. Together, these sections establish the methodological basis for the experimental investigation and the subsequent numerical analysis.

### 2.1. Design of the Temperature-Measuring Tool Holder

**Figure 1:**
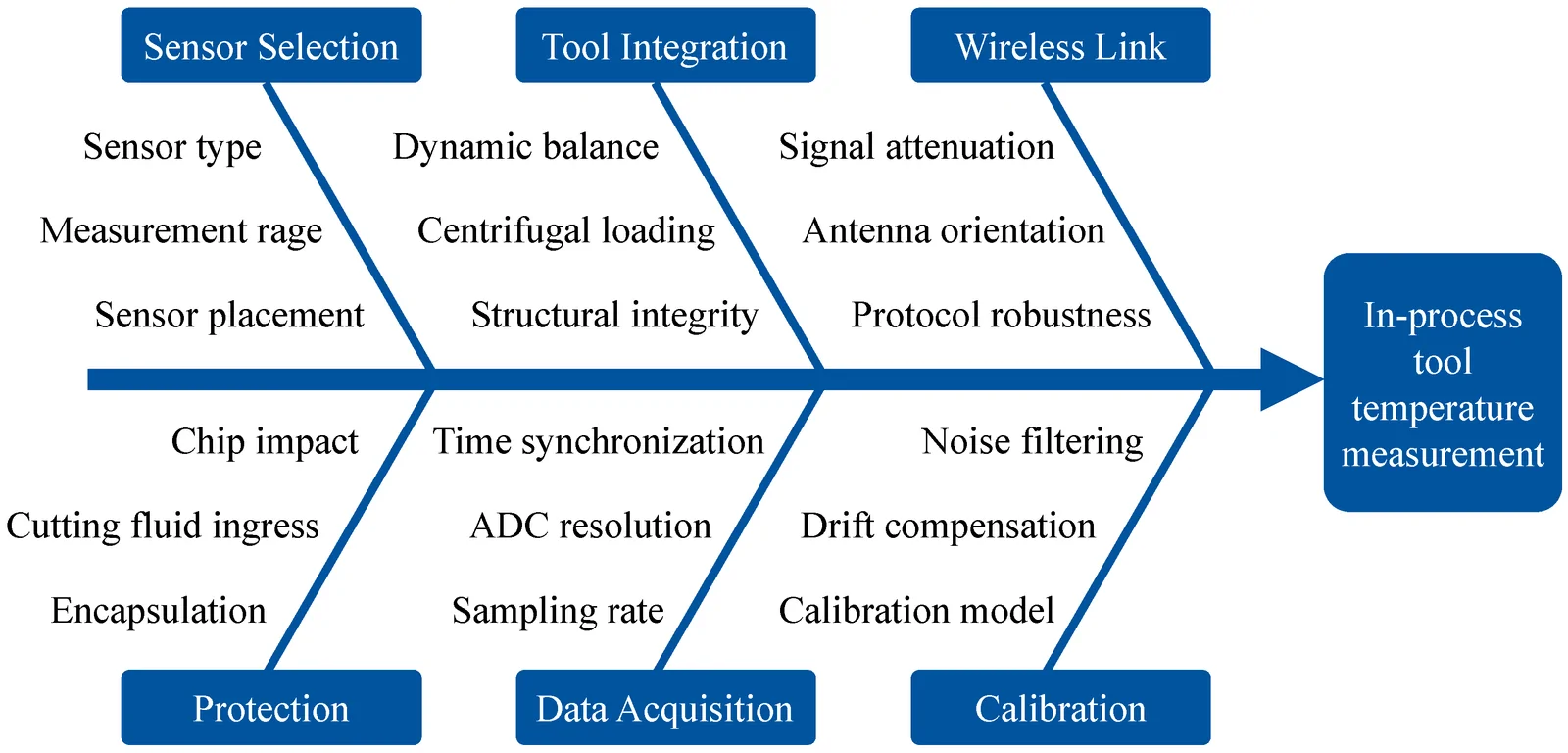
Fishbone analysis of the principal design factors affecting an embedded milling-cutter temperature sensor.

Temperature measurement in milling is challenging because of the harsh process environment and the dynamic behavior of the rotating cutting tool. To establish a systematic and application-oriented design framework, a fishbone analysis was conducted to identify the principal factors influencing the performance of an embedded tool-temperature measurement system. As shown in Figure 1, six interrelated design domains were considered.

The identified design domains encompass sensor selection and positioning, mechanical integration, wireless communication, environmental protection, data acquisition, and signal processing. The sensing principle, measurement range, and sensor position determine the system’s ability to capture rapid temperature variations near the cutting edge with minimal thermal delay. At the same time, the integration of the sensor and electronic components should preserve the structural integrity and dynamic balance of the cutter under rotational and centrifugal loading. Because direct cable transmission is impractical for a rotating tool, wireless communication was adopted, with particular attention paid to signal attenuation caused by the surrounding metallic components and to transmission reliability. The electronics also require a compact and mechanically robust enclosure to protect them against cutting-fluid ingress and chip impact. In addition, the sampling frequency, analog-to-digital resolution, and temporal synchronization of the acquisition system should be sufficient to resolve the transient thermal behavior associated with intermittent milling. Calibration, drift compensation, noise suppression, and electromagnetic-interference filtering are further required to ensure stable and traceable measurements under varying operating conditions.

Based on these design considerations, an embedded temperature measurement system was developed for integration into a rotating milling cutter. A Type-K thermocouple supplied by TC Mess- und Regeltechnik GmbH (Mönchengladbach, Germany) was selected, offering a measurement range of 0 to 1100 °C. The thermocouple has a diameter of 0.2 mm and a thermal response time of approximately 15 ms. The thermal response time characterizes the dynamic behavior of the thermocouple, while the ADC sampling rate determines the temporal resolution of the electronic data acquisition system. The low-level thermoelectric voltage was conditioned by an AD8495 analog-output Type-K thermocouple amplifier (Adafruit Industries, New York, NY, USA) [17]. Its analog voltage was digitized by the analog-to-digital converter (ADC) of a Seeed Studio XIAO ESP32C3 development board (Seeed Studio, Shenzhen, China), which integrates an Espressif ESP32-C3 system-on-chip (SoC) [18]. During the cutting experiments, the ESP32-C3-based system acquired temperature data at a sampling frequency of 1 kHz. The sampling rate is controlled by the acquisition firmware, with the continuous analog output of the AD8495 digitized by the ESP32-C3’s integrated ADC. The development board was selected for its compact size, low mass, programmability, and integrated Wi-Fi connectivity, enabling efficient integration into the rotating milling cutter. The integrated ADC further simplifies the hardware architecture by reducing the need for additional electronic components. The measurement performance of the assembled acquisition chain is evaluated through electrical characterization.

The Adafruit AD8495 module includes a nominal 1.25 V reference offset and provides a temperature sensitivity of 5 mV per degree Celsius [19]. Its nominal transfer relation is

(1) $$V_{\mathrm{out}}=1.25\;V+S\;T,\;S=0.005\;V/{}^{\circ}C,$$

where *T* is the indicated thermocouple temperature in degrees Celsius. In the implemented system, the AD8495 amplifier was powered directly by a battery with a nominal voltage of 3.7 V. Its analog output was connected directly to the XIAO D0/A0 pin (GPIO2, ADC1 channel 2) without an external voltage divider [18]. The amplifier and microcontroller shared a common ground, while the microcontroller board was powered through its rear BAT pad.

The ESP32-C3 integrates a 12-bit successive-approximation analog-to-digital converter (ADC), with specified effective input ranges of 0–0.75, 0–1.05, 0–1.30, and 0–2.50 V for attenuation settings ATTEN0 through ATTEN3, respectively [20]. For the direct connection to the AD8495 amplifier, the temperature range of 175–235 °C observed under dry cutting corresponds to a nominal output voltage of 2.125–2.425 V. Accordingly, ATTEN3 was selected to accommodate the required signal range. Based on the nominal 2.50 V input span and 12-bit ADC resolution, the theoretical voltage resolution is approximately 0.61 mV per code, corresponding to a temperature increment of approximately 0.12 °C per code at the nominal amplifier sensitivity. With the standard amplifier offset of 1.25 V and direct signal acquisition, the nominal upper measurement limit of the configured acquisition system is approximately 250 °C.

Previous studies have reported ADC nonlinearity and application-dependent measurement errors in ESP32-based acquisition systems [21,22]. The magnitude of these errors depends on the specific microcontroller variant, input voltage range, and signal acquisition configuration. For the ESP32-C3, the manufacturer specifies a maximum absolute total error of 35 mV within the effective ATTEN3 input range after hardware and software calibration [20]. To evaluate the measurement accuracy of the implemented acquisition system, electrical verification was performed using a Fluke 714 thermocouple calibrator (Fluke Corporation, Everett, WA, USA) after system assembly. This procedure evaluates the accuracy of the electronic signal conditioning and acquisition chain by applying reference thermocouple signals. The verification results are presented in Section 4.1.

**Figure 2:**
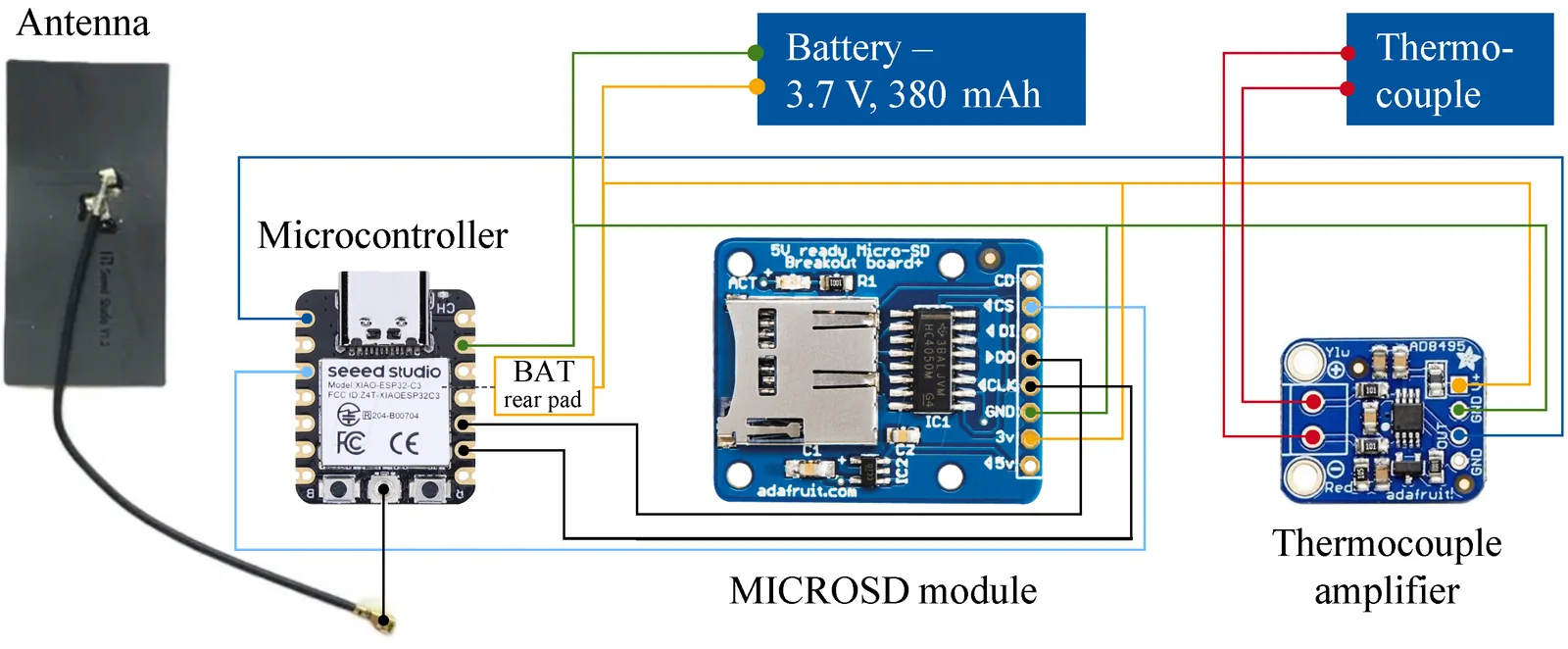
Schematic interconnection and relative dimensions of the electronic components used in the embedded temperature measurement system.

The acquired temperature data are stored locally on a DEBO MICROSD module from Adafruit Industries, LLC (New York, NY, USA). Local storage decouples data recording from wireless network availability. Sampling continuity was checked by examining the recorded timestamps, and no missing samples were identified in the experimental microSD records. Wi-Fi is therefore used primarily for system configuration, status monitoring, and data transfer, whereas computationally intensive signal processing is performed on an external computer. Figure 2 illustrates the interconnection and relative dimensions of the electronic components. The components are shown approximately to scale, with the DEBO MICROSD module being the largest, measuring 32×25×4mm.

**Figure 3:**
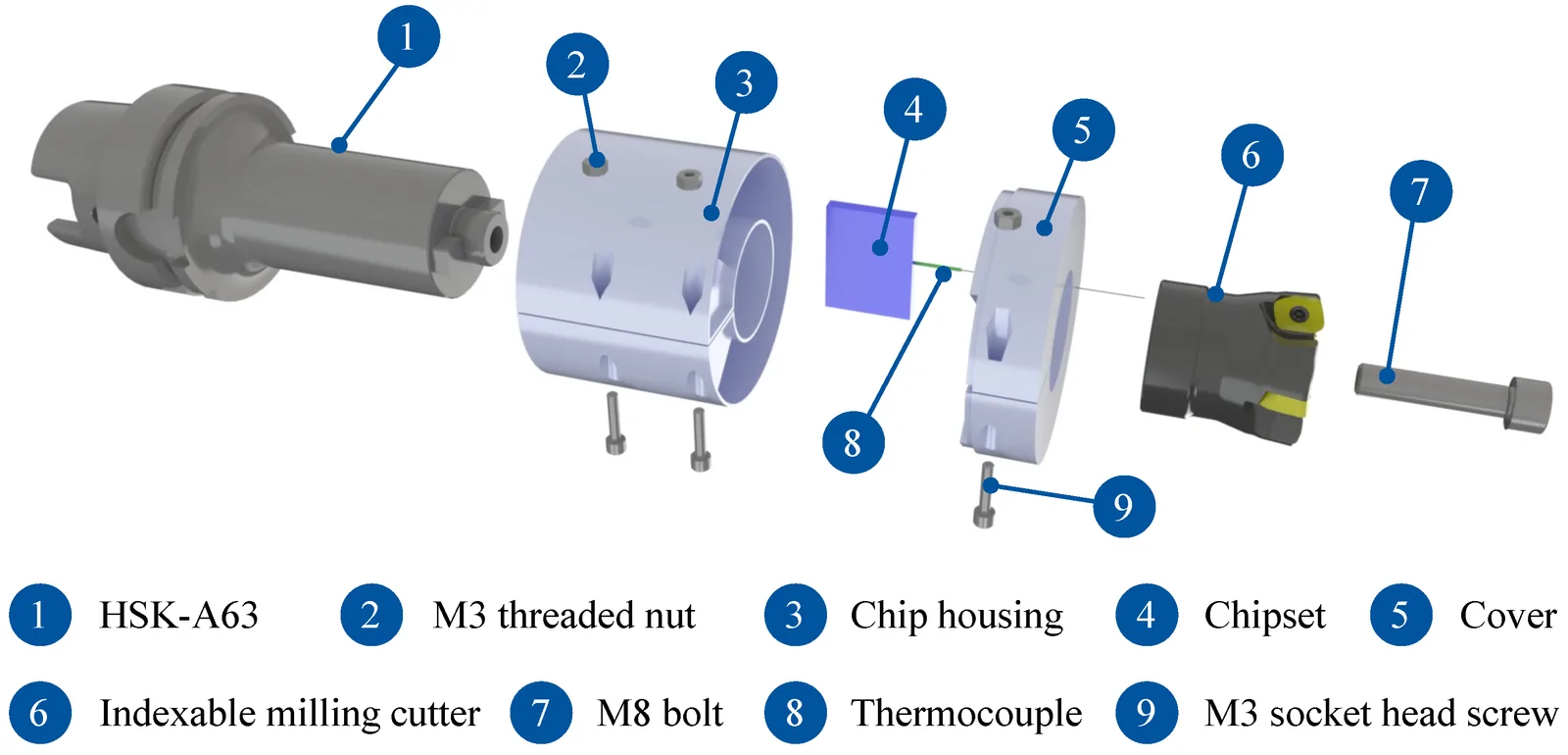
Assembly of the HSK-A63 milling tool holder with the integrated temperature measurement system and its main components.

A high-feed indexable face-milling cutter (MH20-R040Q16-08M, Sandvik Coromant AB, Sandviken, Sweden) mounted on a hollow-shank taper (HSK)-A63 tool holder was selected as the test tool. The HSK-A63 interface is widely used in industrial machining, while high-feed milling cutters generally provide high process stability. This configuration was therefore chosen to minimize the influence of tool dynamics and process-induced vibrations on the temperature measurements. To integrate the sensing and data-acquisition components without modifying the geometry of the tool holder itself, a hub-shaped housing was mounted around the HSK tool holder, as shown in Figure 3. The lightweight housing was additively manufactured from polylactic acid (PLA) and accommodates the electronic components. The thermocouple was routed through a small feedthrough opening in the housing cover and embedded in the cutting insert. The sealed enclosure protects the electronics against cutting-fluid ingress, contamination, and chip impact. The position and mass distribution of the sensing module were selected to minimize its influence on the dynamic balance of the rotating tool, while the low mass of the housing reduces the centrifugal loads acting on both the enclosure and the integrated components at elevated spindle speeds.

Table 1 presents publicly available reference prices for the three core electronic modules as of 24 October 2026, expressed in EUR per unit excluding VAT and shipping. The VAT-inclusive price of the microSD breakout was adjusted using the applicable 19% VAT rate. With a combined reference cost of EUR 28.41, these readily available components demonstrate the affordability of the proposed acquisition architecture. Additional costs associated with the thermocouple, power supply, memory card, wiring, enclosure, assembly, and calibration are not included in this component-level assessment.

### 2.2. Experimental Setup for the Cutting Test

The milling experiments were conducted on 42CrMo4 steel in the quenched-and-tempered condition using cemented-carbide cutting inserts. An MH20-080425M-M20 insert in grade 4340 from Sandvik Coromant AB (Sandviken, Sweden) was employed. The insert features a chemical-vapor-deposition (CVD)-applied $\mathrm{TiCN}/{\mathrm{Al}}_{2}O_{3}/\mathrm{TiN}$ coating and is designed for machining materials in International Organization for Standardization (ISO) groups P, including steels and cast steels, and M, comprising stainless steels. The insert has a nominal corner radius of 2.1 mm. Its microscopic cutting-edge radius was measured using an optical MicroCAD system from LMI Technologies GmbH (Teltow, Germany) and was determined to be 39 ± 0.5 μm.

**Figure 4:**
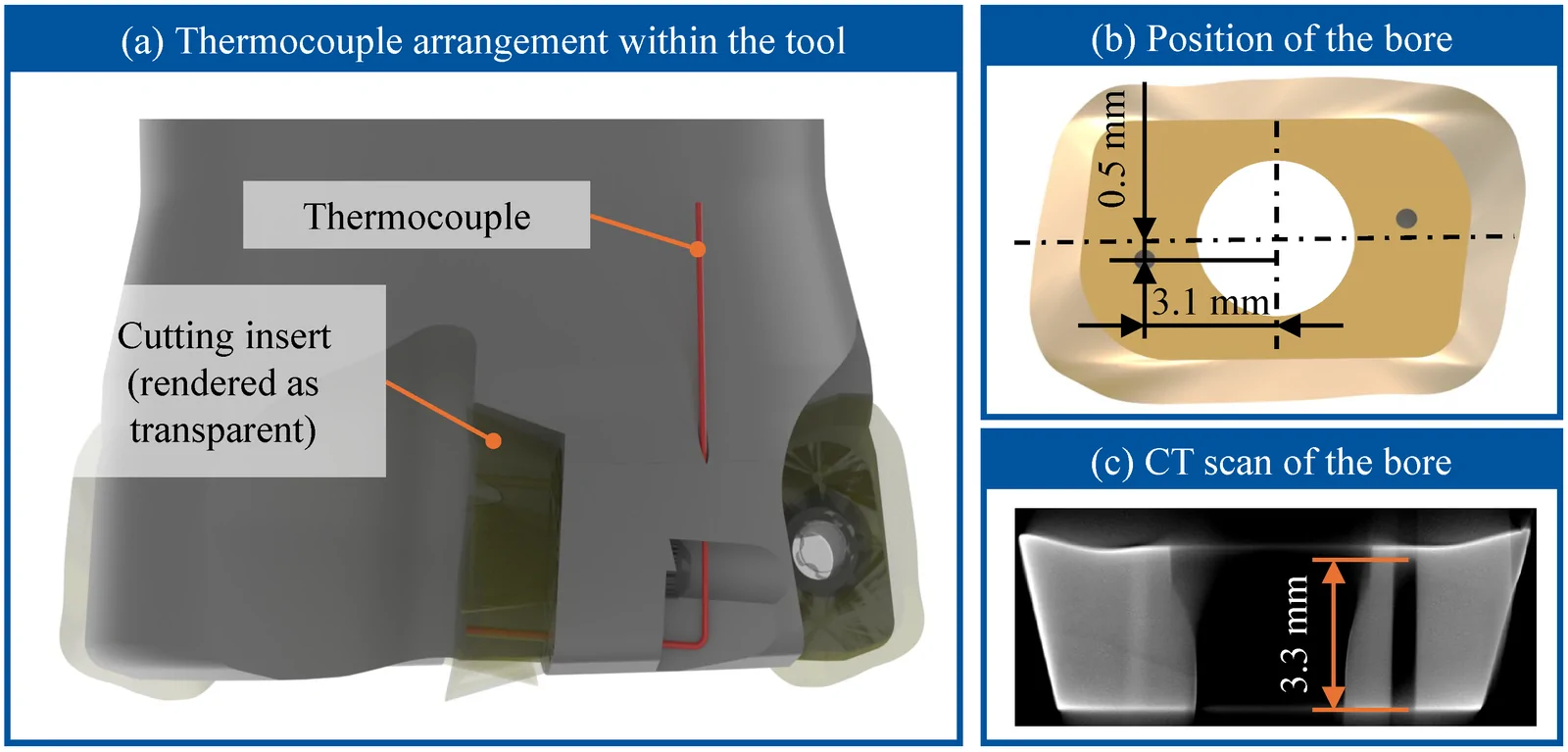
Thermocouple bore arrangement in the cutting tool and its computed-tomography (CT) characterization.

To position the thermocouple within the cutting insert, aligned holes with a diameter of d=0.5mm were produced in the rear surface of the insert and in the tool holder by sinking electrical discharge machining. The resulting thermocouple arrangement within the insert and the milling tool is illustrated in Figure 4. The depth of the hole was selected to position the thermocouple junction as close as possible to the cutting corner while maintaining a minimal distance from the rake face. For this purpose, the centerline of the hole was located 0.5 mm from the insert edge, with the thermocouple junction positioned approximately 150–200 μm beneath the rake face.

Because the precise measurement position has a decisive influence on the interpretation of the tool-temperature data, the location and depth of the thermocouple holes in all inserts were inspected by computed tomography (CT) before the cutting experiments. A representative result is shown in Figure 4c. The inspection confirmed that the variation in hole depth remained within ±0.05 mm. To ensure reliable thermal contact between the thermocouple junction and the bottom of the hole, a thermally conductive paste was applied before installation. The thermocouple wire was additionally secured to the tool holder using a fast-curing adhesive to prevent displacement or withdrawal from the hole under centrifugal loading during tool rotation.

**Figure 5:**
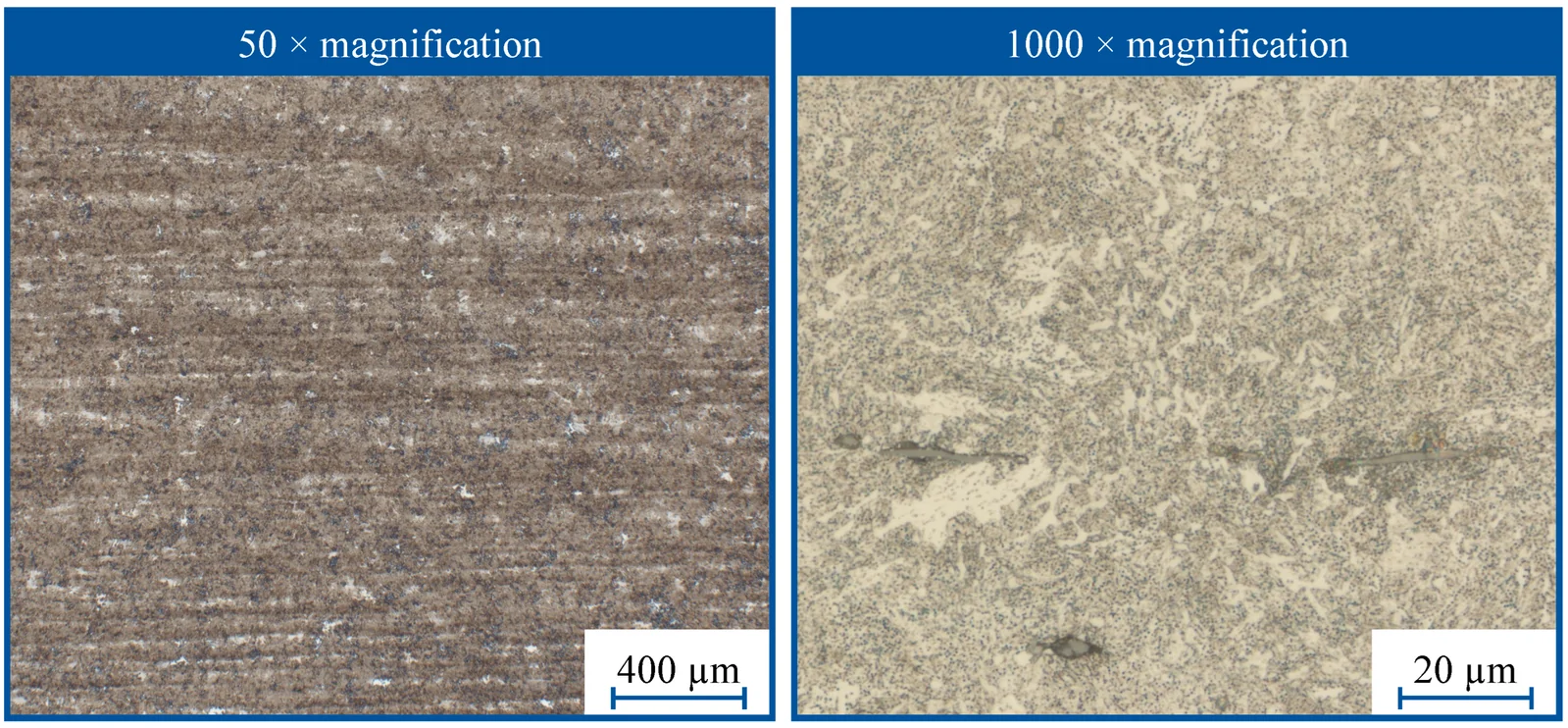
Microstructure of the quenched-and-tempered (QT) 42CrMo4 workpiece at 50× and 1000× magnification.

42CrMo4 steel in the quenched-and-tempered condition was selected as the workpiece material because it is widely used for mechanically and thermally loaded engineering components and therefore offers high industrial relevance. The chemical composition of the material was determined by spark optical emission spectroscopy and is summarized in Table 2. The workpiece had a rectangular geometry with dimensions of 150×150×100mm. Prior to the cutting experiments, metallographic samples were taken from several locations to assess the homogeneity of the workpiece material. The microstructural analysis revealed a uniform phase distribution throughout the investigated regions, as shown in Figure 5. In addition, Vickers hardness measurements were performed at different positions on the workpiece, resulting in an average hardness of 230 ± 5 HV30.

**Figure 6:**
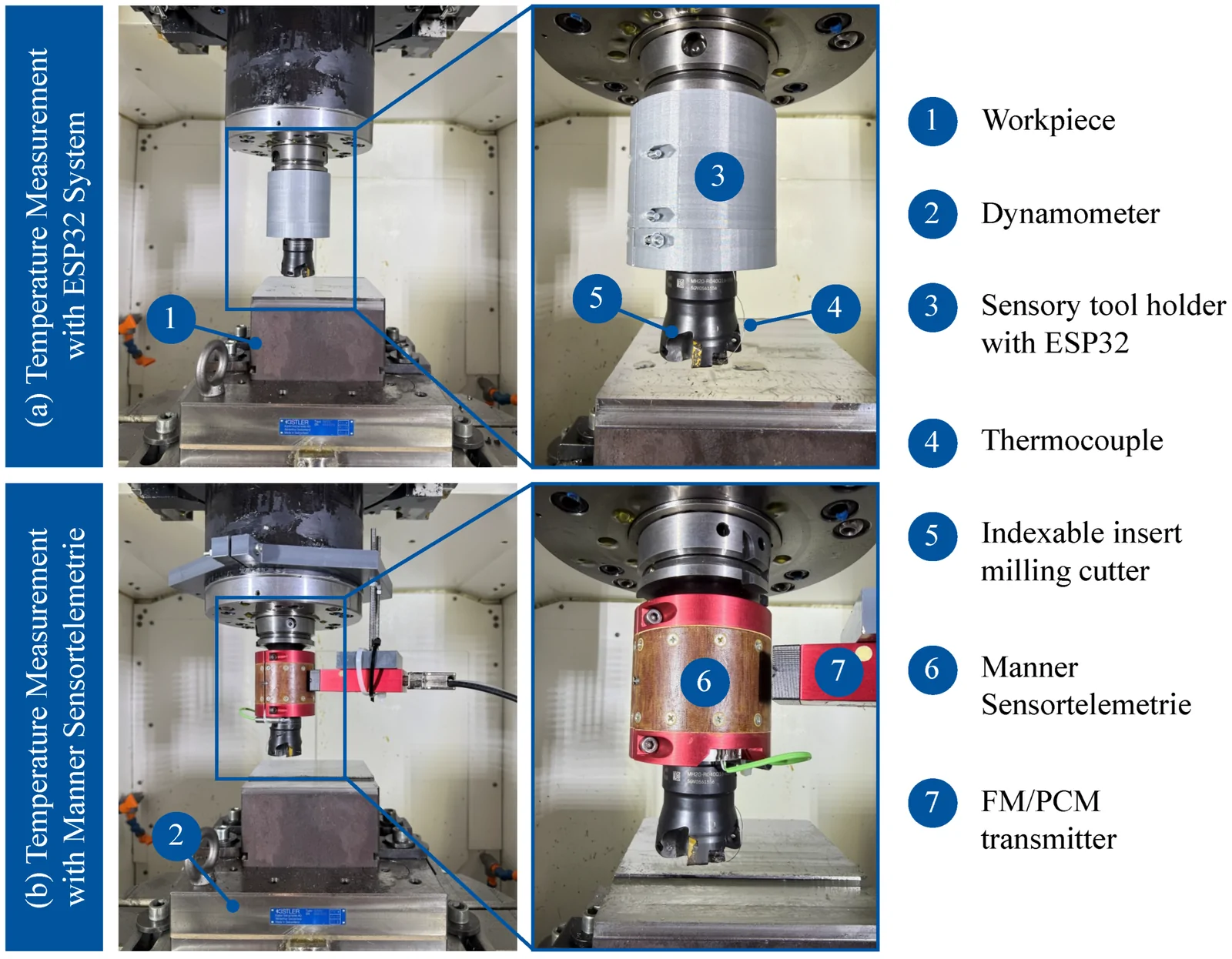
Experimental setups for face-milling temperature measurements using (**a**) the developed ESP32-C3-based system and (**b**) the commercial MANNER telemetry system. FM and PCM denote frequency modulation and pulse-code modulation, respectively.

The cutting experiments consisted of single-axis face-milling tests conducted on a Mazak VARIAXIS i-600 machining center. The workpiece was mounted on a Kistler 9255C dynamometer from the Kistler Group (Winterthur, Switzerland), and the cutting-force components along the three machine-coordinate axes were recorded at a sampling frequency of $f_{s,F}$ = 5 kHz. Figure 6a shows the installation of the ESP32-C3-based temperature measurement system developed in this study.

For independent benchmarking of the developed measurement system, a commercial rotary telemetry system from MANNER Sensortelemetrie GmbH (Spaichingen, Germany) was employed, as shown in Figure 6b. The MANNER system features a hub-shaped configuration with overall dimensions comparable to those of the developed system and consists of a rotating transmitter (rotor) and a stationary receiver (stator). The rotor was mounted around the HSK-A63 tool holder, with the thermocouple connected directly to the rotor. The thermoelectric signal was acquired at a sampling frequency of $f_{s,T}=6620\;\mathrm{Hz}$ and conditioned to an output-voltage range of ±10V for transmission and subsequent acquisition. The signal was transmitted to the stator at a carrier frequency of $f_{\mathrm{trans}}=13.56\;\mathrm{MHz}$ and subsequently acquired using a National Instruments (NI) 9215 data-acquisition module (Austin, TX, USA), with data recording performed in LabVIEW. Prior to the cutting experiments, the rotor output was verified and calibrated using a Fluke 714 thermocouple calibrator. The ±10V signal range applies exclusively to the MANNER–NI reference chain and is not used in the AD8495–ESP32-C3 acquisition system. Consequently, the ADC conversion errors of the ESP32-C3 are not subject to amplification through scaling to this voltage range. The MANNER system provides high-frequency data acquisition and continuous signal transmission. However, the exposed thermocouple connection and the requirement for a stationary stator near the rotating tool restrict its applicability under flood-cooling conditions. Therefore, the MANNER system was used exclusively for dry-cutting experiments. The ESP32-C3-based and MANNER systems were evaluated in separate cutting trials under matched parameters using the same milling tool and thermocouple installation to maintain consistent sensor positioning and thermal contact. The comparison therefore includes cutting-pass variability as well as measurement-system differences.

The milling cutter was equipped with only two of the four available inserts. One insert was instrumented for temperature measurement, while the second was mounted in the diametrically opposite position to maintain the dynamic balance of the rotating tool. Compared with a fully equipped cutter, this configuration increased the number of engagements and the cumulative in-cut time of each insert over a constant feed-path length. All experiments were conducted under down-milling conditions. The cutting parameters used in the experiments are summarized in Table 3. The effects of cutting speed and feed per tooth were investigated while maintaining a constant axial depth of cut of $a_{p}$ = 3 mm and a constant radial width of cut of $a_{e}$ = 20 mm. The parameter combinations were selected such that each cutting condition resulted in a distinct material removal rate, thereby enabling its influence on tool-temperature development to be evaluated. Two trials were performed for each cutting condition to assess the repeatability and reliability of the experimental results. For the flood-cooling experiments, cutting fluid was supplied at a flow rate of 30 L/min through six nozzles arranged around the spindle housing. The direction of each nozzle was adjustable, and the nozzles were directed toward the milling zone at the cutter–workpiece interface.

## 3. Numerical Model and Inverse Calibration for Tool-Temperature Estimation

**Figure 7:**
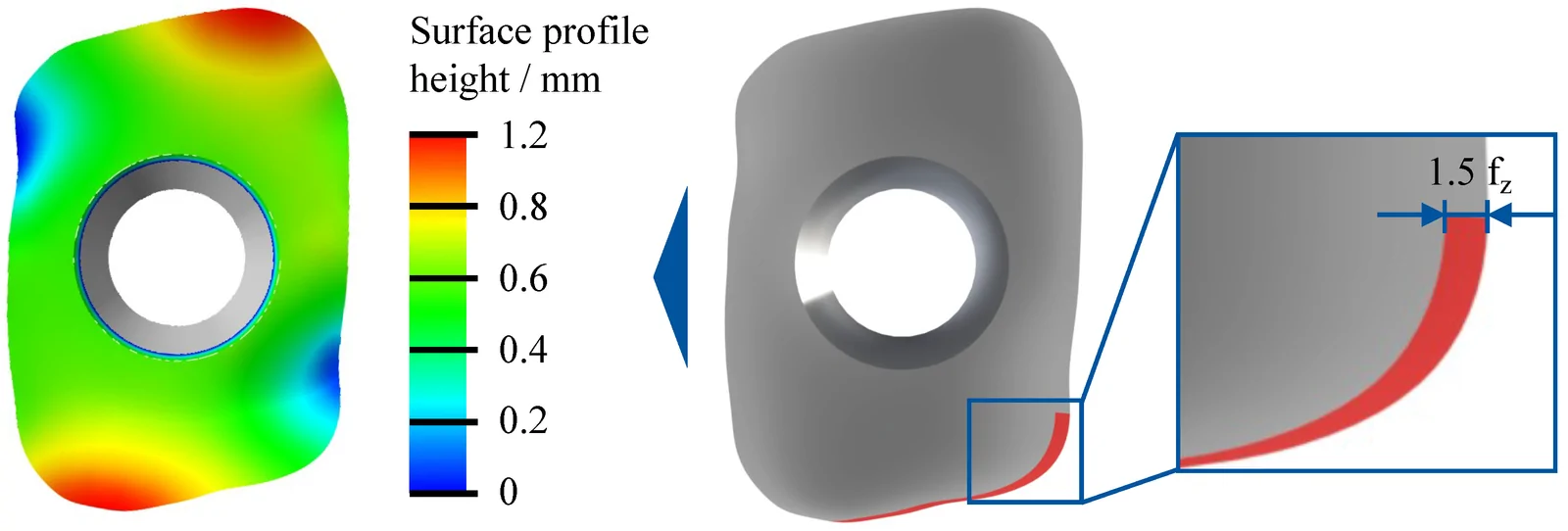
Measured surface-height profile of the cutting insert and definition of the heat-source region based on the undeformed chip cross-section.

Building on the experimental temperature measurements, a finite element model was developed to investigate the effects of the cutting parameters on heat transfer into the tool and the cooling effectiveness of the cutting fluid. The model incorporates the actual three-dimensional geometry of the cutting insert and was used, through inverse calibration, to estimate the effective heat input into the tool and the resulting transient temperature field. The insert geometry was measured using an InfiniteFocus G5 optical measurement system from Bruker Austria GmbH (Raaba, Austria) and subsequently imported in Standard for the Exchange of Product Model Data (STEP) format into the commercial finite element software ABAQUS 2021, in which the thermal model was established. Figure 7 shows the measured surface-height profile of the rake face.

### 3.1. Governing Equation and Heat-Source Representation

Let Ω denote the three-dimensional insert domain and T(x,t) its temperature at position x and time *t*. Heat transfer within the insert is described by transient conduction, with cutting heat supplied through the surface rather than through a volumetric source:

(2) $$\rho c_{p}\left(T\right)\frac{\partial T}{\partial t}=\nabla \;\cdot \;\left[k(T)\nabla T\right],\;x\in \Omega .$$

Here, ρ is the density, $c_{p}\left(T\right)$ is the specific heat capacity, and k(T) is the thermal conductivity. The initial condition is written as

(3) $$T\left(x,0\right)=T_{0}\left(x\right).$$

The function $T_{0}\left(x\right)$ denotes the prescribed initial temperature field. A spatially uniform initialization is the special case $T_{0}\left(x\right)=T_{0}$. The initial field, the thermal properties, and the boundary conditions together determine the subsequent thermal response.

During machining, heat is generated primarily in the primary shear zone, at the tool–chip interface, and at the tool–workpiece interface. Heat enters the cutting tool predominantly through the tool–chip contact region because of the high interfacial temperature and the direct contact between the chip and the rake face. Following an empirical approximation used in earlier thermal cutting models, the tool–chip contact length was set to 1.5 times the undeformed chip thickness [26]. Because contact length depends on the tool, workpiece, and cutting condition, this value is treated as a modeling assumption rather than a directly measured geometry. Although the local heat flux generally decreases from the cutting edge toward the end of the contact region because of variations in friction, contact pressure, and surface topography, a uniform heat-flux distribution was assumed, consistent with simplified thermal-source formulations used in previous tool-temperature models [26,27]. In the present geometry, the assumed contact length was less than 0.6 mm, compared with an insert width of 8 mm, and the analysis focused on the total heat input rather than local variations within the contact region. The heat-source region was defined on the basis of the undeformed chip cross-section and is highlighted in red in Figure 7.

Because milling is an interrupted cutting process, heat was applied to the insert only during engagement with the workpiece. Under down-milling conditions, the undeformed chip thickness decreases continuously from its maximum value at tool entry to approximately zero at tool exit. Consequently, the instantaneous heat input would also be expected to vary during each engagement. In the present model, however, the heat flux was assumed to remain constant over the engagement interval and was set to zero during the non-engagement period. For the investigated cutter geometry and radial width of cut, each insert was engaged over approximately 90°, corresponding to 25% of one tool revolution. Owing to this comparatively short engagement time, particularly at the investigated spindle speeds, the temporal variation of the heat input within a single engagement was neglected. This assumption reduced the model complexity while maintaining the prescribed cycle-integrated heat input within the model.

Spatially uniform heat sources were also adopted as practical approximations for inverse tool-temperature analysis in turning and side milling [15,16]. These studies justified spatial averaging by the small contact region relative to the tool dimensions, with the milling study additionally considering the short heat-application duration. This precedent provides methodological support for simplified heat-source representations in inverse thermal analysis. Following this approach, the present model assumes a tool–chip contact length of 1.5 times the undeformed chip thickness, a spatially uniform heat-flux distribution, and a constant heat-flux density during each tool engagement.

For a single modeled insert, the rectangular heat-input pulse is expressed using the spindle speed *n* in revolutions per minute, the revolution period $\tau_{r}$, the engagement duration $\tau_{e}$, and the first engagement time $t_{0}$:

(4) $$\begin{array}{c} \tau_{r}=\frac{60}{n},\;\tau_{e}=\frac{\tau_{r}}{4},\\ g\left(t\right)=\left\{\begin{array}{cc} 1, & t\geq t_{0}\;\mathrm{and}\;\mathrm{mod}\left(t-t_{0},\tau_{r}\right)<\tau_{e},\\ 0, & \mathrm{otherwise}. \end{array}\right. \end{array}$$

Using a cutter diameter of D=40 mm to convert cutting speed to spindle speed according to $n=1000v_{c}/\left(\pi D\right)$, with $v_{c}$ in m/min, the cutting speeds of 80, 120, and 160 m/min correspond to approximately 637, 955, and 1273 rpm, respectively. For the assumed 90-degree engagement, Equation (4) gives single-insert engagement times of approximately 23.56, 15.71, and 11.78 ms, respectively. The thermocouple response time of approximately 15 ms is therefore of the same order as these engagement times and exceeds the engagement duration at 160 m/min. Although the electronic acquisition rate of 1 kHz provides a nominal sampling interval of 1 ms, it does not ensure that the complete thermal measurement chain can accurately resolve the amplitude and timing of tooth-engagement-scale temperature fluctuations. Heat conduction and thermal storage within the insert, together with the thermocouple response, attenuate and delay rapid local temperature variations. The measurements are therefore used to characterize the slower internal temperature evolution and calibrate the thermal model, while local cyclic temperature variations near the cutting edge remain model-dependent estimates.

Thus, the same insert receives one heat pulse per revolution. The presence of two inserts on the cutter does not halve this individual-insert period. Denoting the heat-source surface by $\Gamma_{q}$, its area by $A_{q}$, and the constant engagement heat-flux intensity by $q_{0}$, the prescribed inward heat flux and the corresponding heat-input rate are

(5) $$\begin{array}{c} q_{\mathrm{in}}\left(x,t\right)=q_{0}g\left(t\right)\;\left(x\in \Gamma_{q}\right),\\ {\dot{Q}}_{\mathrm{in}}\left(t\right)=\int_{\Gamma_{q}}q_{\mathrm{in}}\;dA=q_{0}A_{q}g\left(t\right). \end{array}$$

The energy supplied to one insert during a complete engagement is consequently $E_{\mathrm{in}}=q_{0}A_{q}\tau_{e}$, and its average heat-input rate over a full revolution is $q_{0}A_{q}/4$. These relations distinguish heat-flux intensity from total heat input.

Additional frictional heat is generated at the interface between the principal flank face and the workpiece. For an unworn cutting edge, however, the corresponding contact area is comparatively small and increases primarily with the progression of flank wear. Since the experiments and simulations were conducted using unworn inserts, heat generation at the flank face was assumed to be negligible relative to the heat transferred through the rake face and was therefore omitted from the model.

### 3.2. Thermal Boundary Conditions

Thermal contact was defined between the cutting insert and the tool holder, with the latter assumed to remain at ambient temperature. Based on the tool-holder material, a thermal contact conductance of $h_{c}$ = 3.8 $\mathrm{kW}\;m^{-2}\;K^{-1}$ was applied at the insert–holder interface [28]. Under dry cutting, free convection was imposed on all exposed tool surfaces, except the heat-source region and the insert–holder interface, using a convective heat-transfer coefficient of $h_{\mathrm{free}}$ = 25 $W\;m^{-2}\;K^{-1}$, following the boundary-condition magnitude used in a prior tool-temperature model [27]. Treating the holder as fixed at ambient temperature is an additional simplifying boundary condition. Holder transients were not independently measured in this study. With n denoting the outward unit normal, the conductive heat flux leaving the insert is $q_{n}=-k\left(T\right)\nabla T\cdot n$. The dry-cutting boundary conditions therefore read

(6) $$-k\left(T\right)\nabla T\cdot n=\left\{\begin{array}{cc} -q_{0}g\left(t\right), & x\in \Gamma_{q},\\ h_{\mathrm{free}}\left(T-T_{\infty }\right), & x\in \Gamma_{\mathrm{free}},\\ h_{c}\left(T-T_{\mathrm{holder}}\right), & x\in \Gamma_{c}. \end{array}\right.$$

The surfaces $\Gamma_{\mathrm{free}}$ and $\Gamma_{c}$ are the exposed surfaces with free convection and the insert–holder contact surface, respectively. They exclude the prescribed heat-source surface in the dry model. The minus sign on the source term denotes heat entering the insert. The ambient temperature is $T_{\infty }$, and the holder temperature is fixed at $T_{\mathrm{holder}}=T_{\infty }$.

Under flood cooling, the effective coolant heat-loss term on the surface assigned the coolant boundary condition, $\Gamma_{\mathrm{cf}}$, is

(7) $$q_{\mathrm{loss},\mathrm{cf}}=h_{\mathrm{cf}}\left(T-T_{\mathrm{cf}}\right),\;x\in \Gamma_{\mathrm{cf}}.$$

Here, $T_{\mathrm{cf}}$ is the cutting fluid reference temperature and $h_{\mathrm{cf}}$ is the fitted effective heat-transfer coefficient. On a surface subject to both cooling and prescribed heating, the net outward flux is the heat-loss term minus $q_{0}g\left(t\right)$. Thus, source loading and convective heat removal enter the same surface energy balance with opposite signs.

The influence of thermal boundary conditions on predicted tool temperatures has also been demonstrated by Winter et al. [29] in side milling. For an identical total heat input, their spatially and temporally averaged heat-flux boundary condition resulted in a maximum temperature approximately 180 K lower than that obtained with spatially and temporally resolved heat loading after 50 tool revolutions. Their findings further indicate that adiabatic boundary assumptions, which neglect heat dissipation to the surroundings and machine structure, can lead to overestimated tool temperatures. These results highlight the importance of heat-source representation and thermal boundary conditions in numerical temperature prediction. Although the reported temperature difference is specific to their investigated configuration, the underlying considerations are relevant to the interpretation of the present face-milling simulations.

In the present inverse thermal analysis, the identified heat input and effective convective heat-transfer coefficient are influenced by the assumed tool–chip contact geometry, heat-flux distribution, and thermal boundary conditions, including heat transfer to the tool holder and surrounding coolant. For example, assuming stronger heat transfer from the insert to the holder would generally require a higher calibrated heat input to reproduce the same internal temperature. Consequently, different combinations of heat input and heat-transfer conditions may yield similar agreement with the measured temperature while producing different local temperature distributions. This potential parameter compensation highlights the importance of the adopted modeling assumptions and motivates further sensitivity analyses to assess the robustness of the reconstructed temperature fields.

For each cutting condition, the effective heat flux and coolant coefficient were identified sequentially using the endpoint calibration described in Section 3.4. Retaining the dry-calibrated heat input under flood cooling is a simplifying assumption motivated by the largely overlapping cutting-force curves at matched cutting conditions, which suggest similar mechanical cutting power. This evidence does not establish identical heat transfer into the tool, since coolant-induced changes in friction, contact conditions, and heat partitioning may still occur. Fixing the heat input also avoids fitting two coupled parameters to a single end-of-cut temperature. Consequently, the fitted cooling coefficient is conditional on the assumed heat input and may compensate for unmodeled changes in that input while yielding different local temperatures. Quantifying this dependence requires varying the prescribed heat input, refitting the coolant coefficient, and independently checking the predicted temperatures. This sensitivity and validation study lies beyond the scope of the present methodological demonstration.

The heat-partition ratio into the tool, β, is calculated by normalizing the inverse-calibrated heat input by the total heat generated during cutting. Assuming that 90% of the mechanical cutting energy is converted into heat, the denominator is $0.9E_{\mathrm{cut}}$. For an evaluation interval *I* associated with the modeled insert, the heat transferred into the tool, the corresponding mechanical cutting energy, and the heat-partition ratio are

(8) $$\begin{array}{cc} E_{\mathrm{tool}} & =\int_{I}\int_{\Gamma_{q}}q_{\mathrm{in}}\left(x,t\right)\;dA\;dt=q_{0}A_{q}\int_{I}g\left(t\right)\;dt,\\ E_{\mathrm{cut}} & =\int_{I}F_{c}\left(t\right)v_{c}\left(t\right)\;dt,\;\beta =\frac{E_{\mathrm{tool}}}{0.9E_{\mathrm{cut}}}. \end{array}$$

Here, $F_{c}\left(t\right)$ is the tangential cutting force in the positive cutting-power convention, obtained by the coordinate transformation in Section 4.2. With cutting speed in metres per second, $F_{c}v_{c}$ is in watts and both integrals are in joules. The numerator is the prescribed inward heat transfer through the source region, not the net energy stored after heat losses.

For one complete engagement at constant cutting speed, with $I=[t_{\mathrm{in}},t_{\mathrm{out}}]$ and $\tau_{e}=t_{\mathrm{out}}-t_{\mathrm{in}}$, the uniform heat-source assumption gives

(9) $$\beta =\frac{q_{0}A_{q}\tau_{e}}{0.9v_{c}\int_{t_{\mathrm{in}}}^{t_{\mathrm{out}}}F_{c}\left(t\right)\;dt}.$$

Both energies must refer to the same insert and time interval. For the two-insert cutter, the force lobe corresponding to the modeled insert is used. Thus, β is an energy ratio, not an average of instantaneous power ratios, and remains conditional on the calibrated source and assumed conversion factor.

In the present study, calibration denotes estimation of these effective parameters from the measured thermocouple record. Because the same record was used for parameter identification, agreement between the simulated and measured temperature at that position constitutes a calibration fit rather than an independent model validation. Consequently, the reconstructed temperature field, local peak temperatures, calibrated effective heat fluxes, and fitted effective convective coefficients are reported as conditional inverse-model estimates.

### 3.3. Discretization and Material Properties

Automatic time incrementation was used in ABAQUS for the transient thermal analysis, with an initial time increment of $1\times 10^{-5}$ s and a maximum time increment of $5\times 10^{-4}$ s. These settings specify the initial increment and the upper increment limit rather than a constant time step.

The cutting insert was discretized using an unstructured tetrahedral mesh consisting of four-node linear heat-transfer elements of type DC3D4. Based on a mesh-sensitivity analysis, a global element size of 0.1 mm was selected, resulting in a total of 1,050,532 elements. This mesh resolution was considered sufficient to capture the steep temperature gradients in the vicinity of the cutting edge [30]. The density of the cutting-insert material was specified as ρ = $1.48\times 10^{4}\;\mathrm{kg}\;m^{-3}$. The density and the temperature-dependent thermal conductivity and specific heat capacity in Table 4 were adopted from prior thermal modeling work for cemented-carbide cutting tools [15,27]. Because the exact substrate composition of the present commercial coated grade may differ from the cited material, transfer of these properties is a source of model uncertainty, particularly above the tabulated conductivity range.

When the simulated temperature exceeded the upper tabulated limit of a material property, that property was held constant at its last tabulated value. Specifically, the thermal conductivity remained at 75.92 $W\;m^{-1}\;K^{-1}$ above 500 °C, and the specific heat capacity remained at 250 $J\;{\mathrm{kg}}^{-1}\;K^{-1}$ above 1500 °C. This treatment is a constant extension of the tabulated properties beyond their available temperature ranges, not a characterization of the material at the reconstructed peak temperatures. No additional temperature dependence was introduced above these limits. The density remained constant throughout the simulation at the value specified above.

### 3.4. Temperature Reconstruction and Inverse Parameter Identification

For the linear tetrahedral discretization, the approximate temperature field is represented by the nodal temperatures $T_{a}\left(t\right)$ and finite element shape functions $N_{a}\left(x\right)$:

(10) $$T_{h}\left(x,t\right)=\sum_{a=1}^{N_{\mathrm{node}}}N_{a}\left(x\right)T_{a}\left(t\right).$$

The subscript *h* identifies the spatially discretized field, and $N_{\mathrm{node}}$ is the number of mesh nodes. The nodal histories follow from the transient heat-conduction problem above. For an ideal point observation at the thermocouple position $x_{\mathrm{TC}}$, the simulated sensor temperature is $T_{h}\left(x_{\mathrm{TC}},t\right)$. More generally, let $S_{\mathrm{TC}}$ denote the extraction of the simulated temperature corresponding to the sensor location:

(11) $$T_{\mathrm{TC}}^{\mathrm{sim}}\left(t\right)=S_{\mathrm{TC}}\left[T_{h}\left(\cdot ,t\right)\right].$$

This distinguishes the internal observation used for calibration from the maximum temperature reconstructed elsewhere in the insert. The extraction operator describes the spatial comparison location and does not itself correct for the thermocouple response time.

Parameter calibration was performed separately for each cutting condition using the temperature at the end of cutting. Let *j* identify a cutting condition and let $T_{\mathrm{end},j}$ denote the temperature at the thermocouple position at the end of that cutting interval. The differences used for the dry and flood-cooling comparisons are expressed as

(12) $$\begin{array}{cc} \delta T_{\mathrm{end},j}^{\mathrm{dry}}\left(q_{0,j}\right)\; & =T_{\mathrm{end},j}^{\mathrm{sim},\mathrm{dry}}\left(q_{0,j}\right)-T_{\mathrm{end},j}^{\mathrm{meas},\mathrm{dry}},\\ \delta T_{\mathrm{end},j}^{\mathrm{flood}}\left(h_{\mathrm{cf},j}\right)\; & =T_{\mathrm{end},j}^{\mathrm{sim},\mathrm{flood}}\left({\hat{q}}_{0,j},h_{\mathrm{cf},j}\right)-T_{\mathrm{end},j}^{\mathrm{meas},\mathrm{flood}}. \end{array}$$

First, $q_{0,j}$ is adjusted to reduce the magnitude of the dry-cutting temperature difference and obtain ${\hat{q}}_{0,j}$. This value is then held fixed while $h_{\mathrm{cf},j}$ is adjusted against the end-of-cut temperature under flood cooling to obtain ${\hat{h}}_{\mathrm{cf},j}$. The hats denote the selected parameter values. The rationale for selecting the end-of-cut temperature and the interpretation of the internal sensor response are discussed in Section 4.3.

The identified parameters represent effective values within the prescribed geometry, material properties, and thermal boundary conditions. The sequential endpoint calibration provides a consistent framework for characterizing heat input and removal, while potential interactions between these mechanisms and the associated parameter uncertainties can be further assessed through sensitivity and identifiability analyses supported by independent temperature measurements.

After parameter calibration, the temperature field is obtained from the same forward thermal problem with the selected parameter values. The temperature fluctuation range is evaluated over the final 0.5 s of cutting, representing the steady cyclic thermal response. Denoting the end of the modeled cutting interval by $t_{\mathrm{end}}$, the evaluation window $I_{\mathrm{ss}}$, the instantaneous maximum over the insert, and its upper and lower limits are defined as

(13) $$\begin{array}{c} I_{\mathrm{ss}}=\left[t_{\mathrm{end}}-0.5\;s,\;t_{\mathrm{end}}\right],\\ T_{\max}\left(t\right)=\max_{x\in \Omega }T_{h}\left(x,t\right),\\ T_{\mathrm{upper}}=\max_{t\in I_{\mathrm{ss}}}T_{\max}\left(t\right),\;T_{\mathrm{lower}}=\min_{t\in I_{\mathrm{ss}}}T_{\max}\left(t\right),\\ \Delta T_{\max}=T_{\mathrm{upper}}-T_{\mathrm{lower}}. \end{array}$$

The upper and lower limits are the maximum and minimum of the instantaneous maximum-temperature history within this final 0.5 s window. The initial heating transient and the subsequent cooling after cutting are excluded from this evaluation. Here, steady cyclic response refers to the approximately stabilized upper and lower temperature envelopes, while tooth-engagement-scale oscillations persist. Thus, $\Delta T_{\max}$ describes the peak-to-peak temperature variation in the final steady cyclic regime rather than the temperature excursion over the entire cutting process. The measured internal temperature, the reconstructed local maximum, and this cyclic temperature range are distinct quantities connected by the conduction model and its inverse calibration.

The reconstructed peak temperatures exceed the tabulated range of thermal conductivity and, under certain conditions, that of specific heat capacity. Their predicted magnitudes depend on the constant extension of material properties described in Section 3.3, as well as the assumed heat-source geometry and thermal boundary conditions. Although a quantitative sensitivity analysis of the high-temperature property assumptions has not been performed, the reconstructed temperature fields provide insight into local thermal gradients and transient heat-transfer behavior within the adopted model. Accordingly, the absolute peak temperatures and their quantitative differences between cutting conditions are interpreted as model-dependent estimates that require independent validation.

## 4. Results and Discussion

The results and discussion are organized into three parts: measurement system verification and calibration, experimental investigations, and numerical thermal analysis. First, the electrical performance of the ESP32-C3-based measurement system is assessed using reference temperature signals, while the voltage–temperature conversion of the commercial MANNER telemetry system is characterized through calibration. These assessments are complemented by a quantitative comparison of the temperature histories recorded by both systems under matched dry-cutting conditions. Subsequently, the experimental results are analyzed to evaluate the influence of cutting parameters and cooling conditions on internal tool temperatures and cutting forces. Finally, the experimental measurements are integrated with a three-dimensional inverse finite element model to reconstruct the transient temperature field within the cutting insert and estimate the effective heat input, cooling behavior, and heat partitioning under the investigated conditions.

### 4.1. Electrical Verification Results

Table 5 summarizes electrical verification over approximately 25–250 °C. The within-record standard deviations ranged from 0.057 to 0.340 °C, while mean biases ranged from −0.359 to +2.449 °C, with the largest deviations between 75 and 150 °C. Pooling all 125,504 recorded temperature readings across the ten reference setpoints yielded an RMSE of 1.523 °C. Each reading was weighted equally when averaging squared deviations from its reference setpoint, so each setpoint contributed in proportion to its sample count.

Following the electrical verification of the ESP32-based system, the MANNER reference system was characterized to establish the voltage–temperature conversion required for subsequent quantitative comparisons. The calibration was based on 57 paired reference values, covering output voltages of 0.181–7.932 V and temperatures of 0.6–702.8 °C. An ordinary least-squares regression, with temperature as the dependent variable and a freely fitted intercept, yielded the following conversion relationship.

(14) $${\hat{T}}_{M}=aU_{M}+b,\;a=90.6814\;{}^{\circ}C\;V^{-1},\;b=-12.8014\;{}^{\circ}C.$$

Here, $U_{M}$ is the MANNER output voltage in volts and ${\hat{T}}_{M}$ is the temperature predicted by the fitted relation. The inverse of this relation is $U_{M}=\left({\hat{T}}_{M}-b\right)/a$, corresponding to an output sensitivity of approximately 11.028 mV °C^−1^. This sensitivity refers to the conditioned MANNER output rather than the raw thermocouple voltage. The root mean square deviation between the fitted and reference temperatures was calculated as

(15) $${\mathrm{RMSD}}_{M}=\sqrt{\frac{1}{N}\sum_{i=1}^{N}{\left(aU_{M,i}+b-T_{\mathrm{ref},i}\right)}^{2}}=2.323\;{}^{\circ}C,\;N=57.$$

The MANNER calibration demonstrated excellent linearity, with a coefficient of determination of $R^{2}=0.999883$ and a maximum absolute residual of 3.868 °C. The calibration range fully covers the temperatures measured during milling, allowing the fitted voltage–temperature relationship to be applied without extrapolation. While the MANNER calibration residual and the ESP32-C3 pooled RMSE characterize the performance of their respective signal-conversion and electronic acquisition chains under controlled verification conditions, additional factors such as thermocouple installation, thermal contact, dynamic response, and machining variability contribute to the overall measurement uncertainty during cutting.

### 4.2. Experimental Analysis of Tool Temperature and Cutting Forces

**Figure 8:**
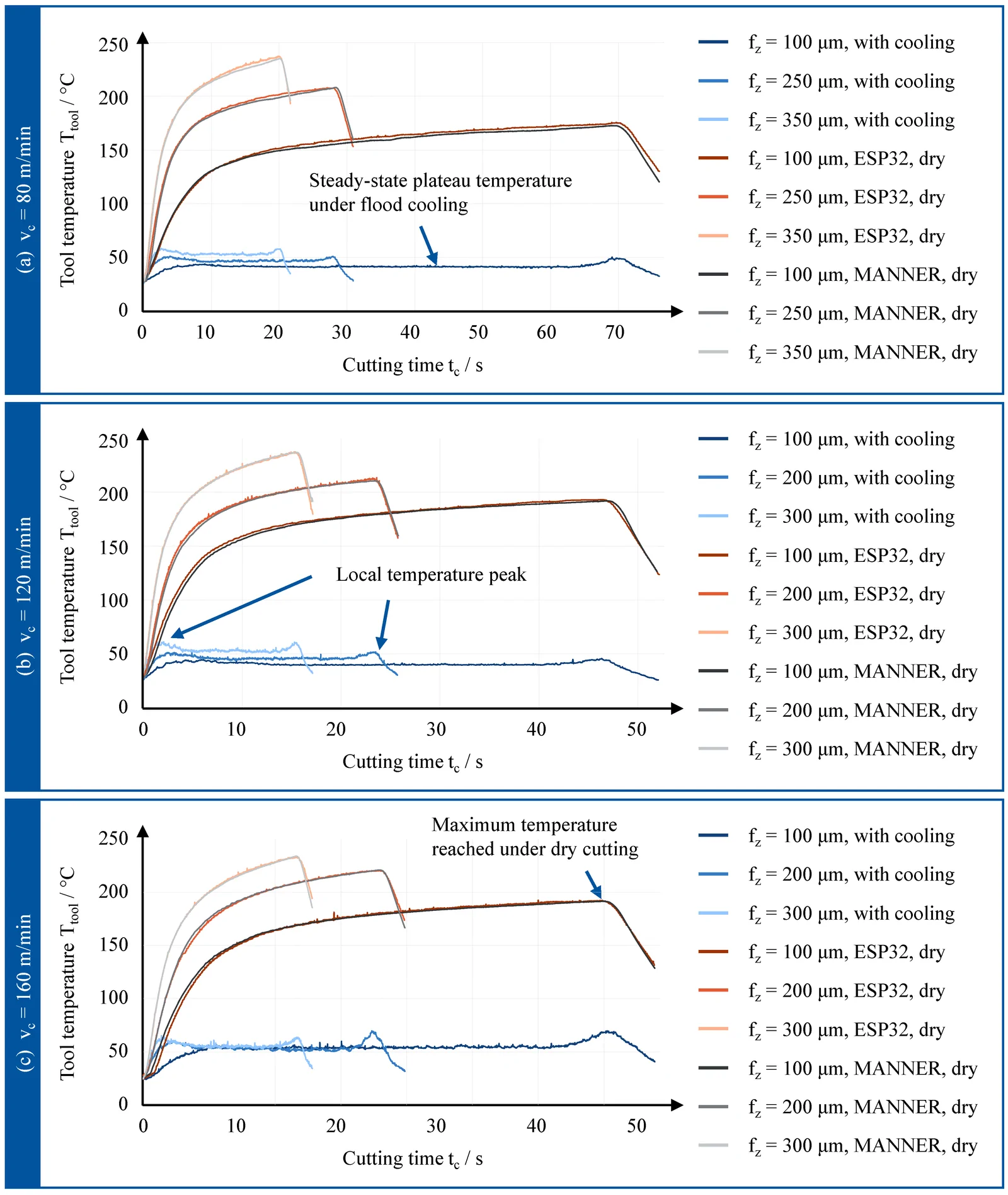
Comparison of tool-temperature evolution measured by the ESP32-based and MANNER telemetry systems under dry and flood cooling conditions at different cutting speeds and feed-per-tooth values.

Figure 8 compares the temperature histories recorded by the ESP32-C3-based and MANNER systems under dry-cutting conditions, with both signals postprocessed using a 350 Hz low-pass filter. Both systems captured similar thermal responses, characterized by a rapid initial temperature increase followed by a more gradual rise due to heat accumulation within the cutting insert. Minor deviations during transient phases may be attributed to differences in acquisition characteristics, signal processing, temporal alignment, and variability between the separate cutting trials described in Section 2.2. The overall agreement supports the consistency of the measured thermal trends, while the ESP32-C3-based system further extends the experimental investigation to flood-cooling conditions.

Dry-cutting temperatures reached approximately 175–235 °C, whereas flood-cooling temperatures generally remained below 70 °C. Under flood cooling, local peaks at tool entry and exit may reflect transient reductions in coolant access and wetting as the engagement geometry changes. This proposed mechanism remains unverified because local fluid coverage and flow were not independently measured.

For quantitative comparison, the temperature measurements obtained from the ESP32-based system and the commercial MANNER system under dry cutting conditions were evaluated using the root mean square deviation (RMSD) and mean signed difference (MSD). Table 6 summarizes the comparison results for the investigated cutting conditions.

Across the nine conditions, RMSD ranged from approximately 1.8 to 3.4 °C, corresponding to range-normalized values of 0.9–2.1%. The largest deviation occurred at $v_{c}=80$ m/min and $f_{z}=250$ μm, and the smallest at $v_{c}=160$ m/min and $f_{z}=300$ μm. Mean signed differences ranged from −0.4 to +2.3 °C. RMSD and MSD describe agreement between separate cutting trials, including process variability, rather than the intrinsic accuracy of either system.

**Figure 9:**
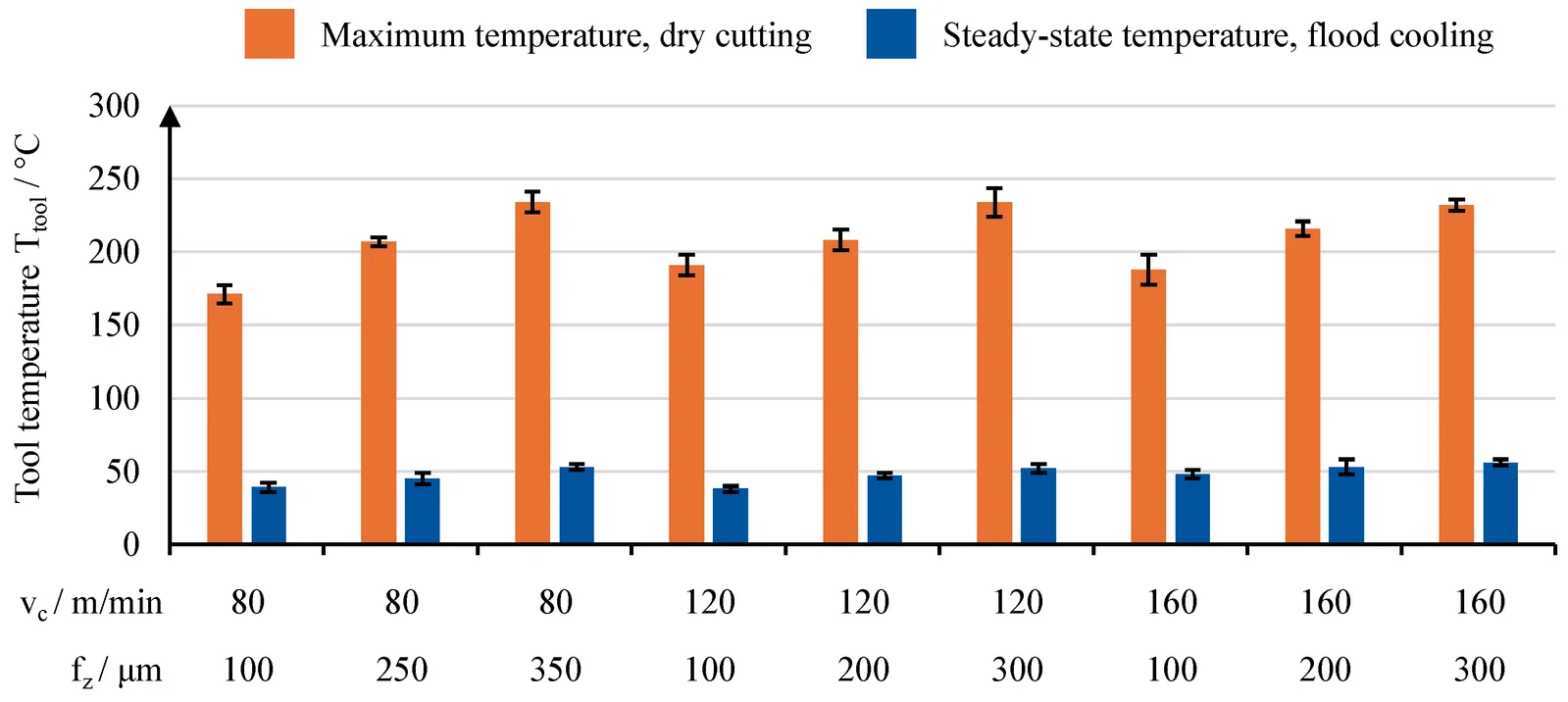
Maximum tool temperature under dry cutting and steady-state tool temperature under flood cooling for different cutting parameters.

To further evaluate the relationship between tool temperature and the cutting parameters, Figure 9 summarizes the maximum tool temperature under dry cutting and the steady-state temperature under flood cooling for all investigated conditions. For each cutting condition, the bar height represents the arithmetic mean of the corresponding temperature values obtained from two repeated experiments (n=2). Under dry cutting, the peak temperature is extracted from each experiment, and the error bar extends from the smaller to the larger of these two peak values. Under flood cooling, the same calculation is applied to the steady-state temperature obtained from each experiment. Thus, the full error-bar span represents the difference between the two experimental values and provides a descriptive measure of between-trial variability.

Under dry cutting, the tool temperature increased consistently with increasing feed per tooth at all cutting speeds. This trend can be attributed to the larger undeformed chip thickness and the associated increase in mechanical and thermal loading during each tooth engagement. Although a higher feed per tooth reduces the total machining time for a given cutting path, the increased heat generation per engagement dominates the thermal response, resulting in higher maximum tool temperatures.

The influence of cutting speed was less pronounced than that of the feed per tooth. Changes in $v_{c}$ produced only comparatively small differences in the maximum tool temperature. This behavior reflects the competing effects associated with increasing cutting speed. While the rate of heat generation generally increases, the engagement time per revolution decreases, thereby reducing the time available for heat transfer from the tool–chip interface into the cutting insert. The measured internal tool temperature therefore results from the combined effects of heat generation, heat partitioning, and transient heat conduction rather than from the heat-generation rate alone. Under flood-cooling conditions, the measured internal temperature showed a weaker dependence on feed per tooth, consistent with enhanced heat removal and reduced thermal accumulation. At $v_{c}=160$ m/min, temperatures remained close to 50 °C across the investigated feed range.

For quantitative context, Espinoza-Torres et al. [10] reported tool-flank temperatures of approximately 304–518 °C during dry up-milling of AISI 4140 steel at $v_{c}$ = 300–400 m/min and $f_{z}$ = 80–160 μm. The present embedded sensor measured lower internal insert temperatures of 175–235 °C at $v_{c}$ = 80–160 m/min. Because the workpiece condition, speed range, sensor location, and measurement principle differ, this comparison establishes only a literature scale and is not a validation of either temperature field.

In addition to the tool-temperature measurements, the cutting forces were analyzed to provide further insight into the thermal and tribological behavior of the milling process. The tangential cutting force is directly related to the cutting power through $P_{c}\;=\;F_{c}\cdot v_{c}$. Since approximately 90% of the mechanical energy introduced into the cutting process is ultimately converted into heat, the cutting force provides an important basis for estimating the total heat generated and, subsequently, for analyzing its partition among the chip, tool, and workpiece. Furthermore, comparison of the cutting forces under dry cutting and flood cooling provides an indication of whether the cutting fluid produces a significant lubricating effect at the tool–chip interface.

Because the dynamometer was fixed to the machine table, the force components $F_{x}$ and $F_{y}$ were measured in the stationary machine coordinate system, whereas the tangential cutting force $F_{c}$ acts in a direction that continuously changes with the angular position of the rotating cutting edge. A coordinate transformation was therefore required. The tool-entry position was selected as the angular reference. The time $t_{0}$ at which $F_{x}$ and $F_{y}$ began to deviate from their unloaded baseline was identified as the instant of cutting-edge engagement and assigned an angular position of $\phi \;=\;0^{\circ }$. The instantaneous angular position was then calculated from the spindle speed n as

(16) $$\varphi \left(t\right)=\frac{2\pi n}{60}\left(t-t_{0}\right),$$

where n is given in $\min^{-1}$. The measured force vector was subsequently projected onto the instantaneous tangential direction according to

(17) $$F_{c}\left(t\right)=-F_{x}\left(t\right)\sin\theta \left(t\right)+F_{y}\left(t\right)\cos\theta \left(t\right),$$

with

(18) $$\theta \left(t\right)=\theta_{0}+\varphi \left(t\right),$$

where $\theta_{0}$ denotes the angular offset between the positive x-axis of the dynamometer coordinate system and the radial direction of the cutting edge at tool entry. This transformation allows the tangential force component acting along the instantaneous cutting direction to be evaluated over each tooth engagement independently of the fixed orientation of the dynamometer coordinate system.

**Figure 10:**
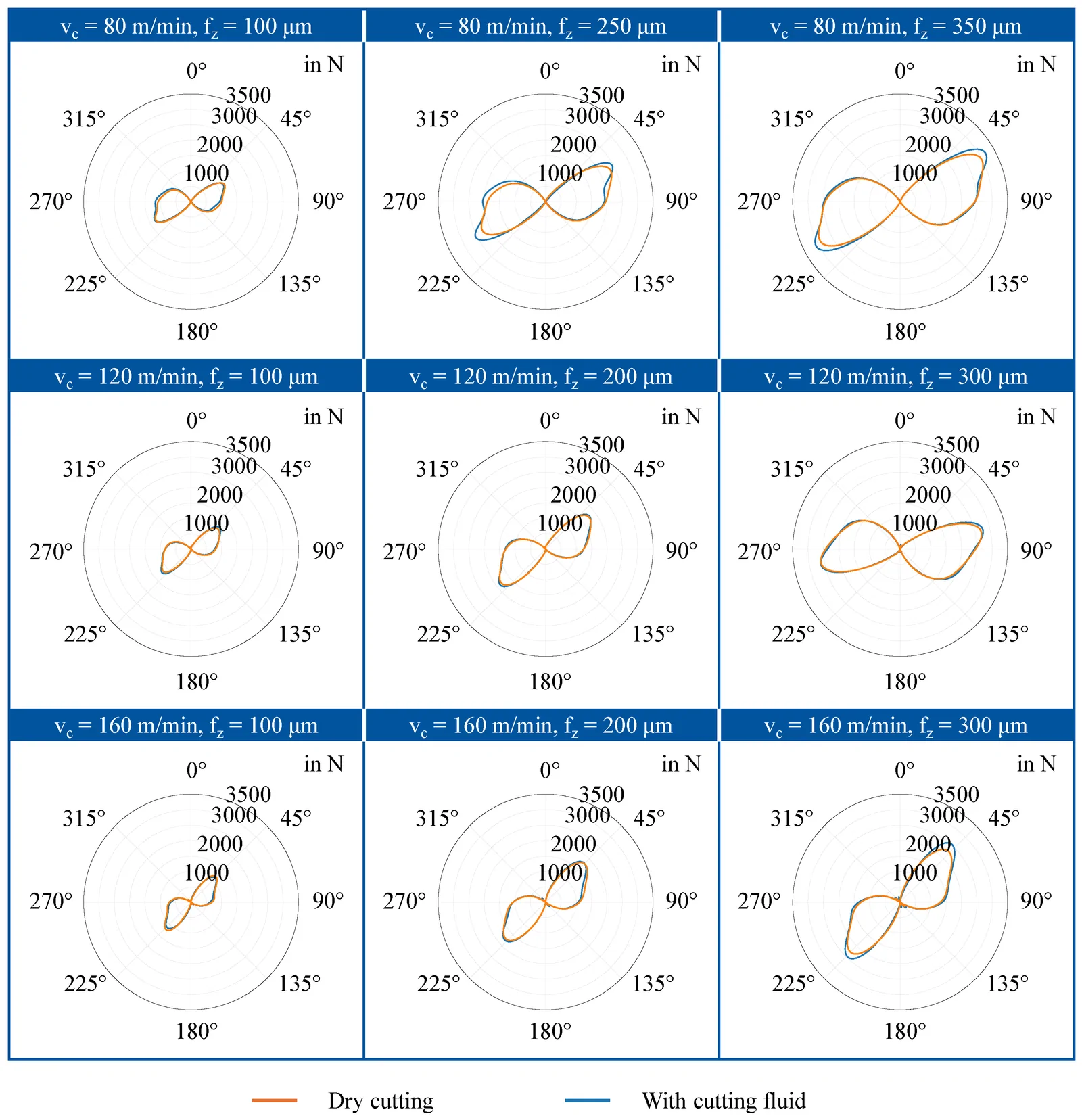
Polar diagrams of the cutting force over one tool revolution for different cutting speeds and feed-per-tooth values under dry cutting and flood cooling.

Figure 10 presents the cutting-force distributions over one tool revolution for the investigated cutting parameters under dry cutting and flood cooling. The polar diagrams exhibit two characteristic force lobes within each revolution, corresponding to the engagement of the two inserts mounted approximately $180^{\circ }$ apart. Among the investigated cutting parameters, the feed per tooth had the most pronounced influence on the cutting-force magnitude. At all cutting speeds, increasing $f_{z}$ resulted in a substantial increase in the maximum cutting force and in the area enclosed by the polar force curve. This behavior is attributed primarily to the increase in undeformed chip thickness and, consequently, in the instantaneous chip cross-sectional area. A larger amount of material must therefore be removed during each tooth engagement, leading to higher mechanical loading of the cutting edge. The increase in force with $f_{z}$ is particularly evident when comparing the lowest and highest feed-per-tooth conditions, whereas the overall shape and angular extent of the force profiles remain similar.

In comparison, the influence of cutting speed on the cutting-force magnitude was considerably less pronounced. For comparable feed-per-tooth values, the force levels remained within a similar range as $v_{c}$ increased. This indicates that, within the investigated cutting-speed range, the mechanical load is governed predominantly by the undeformed chip geometry rather than by cutting speed. Variations in cutting speed may alter strain rate, friction, and thermal softening in the primary and secondary deformation zones, but these effects partly compensate each other and therefore produce only moderate changes in the measured cutting force.

The application of flood cooling produced only limited changes in the cutting-force distributions. For most cutting conditions, the curves obtained under dry cutting and flood cooling largely overlap, and no systematic reduction in force due to the cutting fluid is observed. Local differences occur at individual angular positions, but they are small compared with the force increase caused by a higher feed per tooth. The cutting fluid therefore has a much stronger influence on the thermal response of the cutting insert than on its mechanical loading. This suggests that the cooling effect of the fluid dominates over any reduction in mechanical load caused by lubrication at the tool–chip interface.

### 4.3. Inverse-Calibrated Estimates of Tool Thermal Load and Heat Partitioning

Thermocouple measurements provide the temperature evolution only at a fixed location within the cutting insert and therefore do not directly reveal the overall thermal state of the tool. The inverse-calibrated finite element model described in Section 3 was employed to obtain conditional, model-based estimates of the spatial temperature distribution and thermal loading of the insert. The same analysis was used to derive effective heat-input and heat-partition quantities subject to the adopted modeling assumptions.

**Figure 11:**
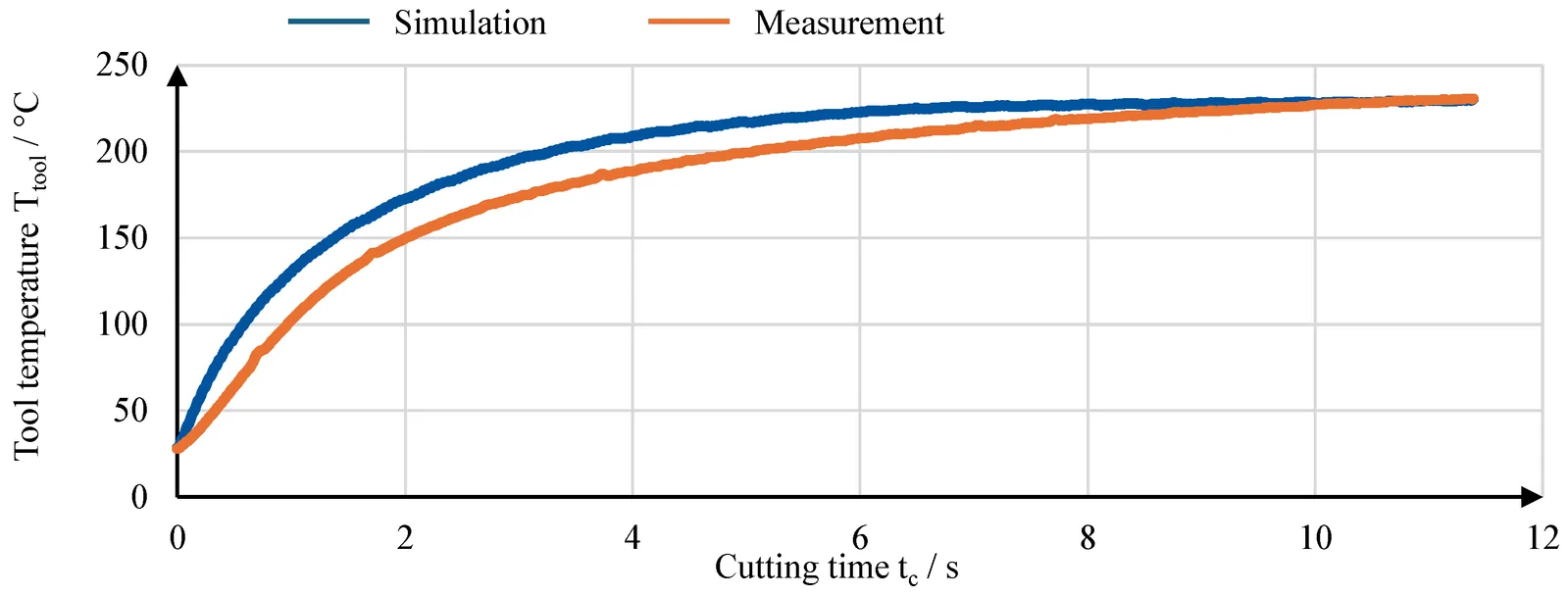
Measured and simulated temperature histories at the thermocouple position after calibration to the end-of-cut temperature for $v_{c}=160$ m/min and $f_{z}=300$ μm.

Figure 11 compares the measured and simulated temperature histories at the thermocouple position for $v_{c}=160$ m/min and $f_{z}=300$ μm after calibration of the heat-source intensity to the measured end-of-cut temperature, as defined in Equation (12). The preceding temperature evolution provides a qualitative assessment of the transient agreement between measurement and simulation. Because the same record is used for calibration and comparison, this assessment is not an independent model validation.

Both the measured and simulated temperature histories exhibit a rapid increase following the onset of cutting, followed by a gradual reduction in the heating rate. The measured temperature rises from approximately room temperature to 230 °C, and the simulated temperature approaches a comparable final level. Near the end of cutting, the sensor temperature changes more slowly than during the initial heating stage. This lower rate of change reduces the sensitivity of the temperature comparison to thermocouple response lag and acquisition delay, providing the rationale for selecting the end-of-cut level for parameter identification. The choice does not eliminate measurement delays, and the agreement at the final temperature reflects the calibration target.

A noticeable deviation occurs during the initial heating stage, where the simulated temperature rises more rapidly than the measured temperature. This difference gradually decreases with increasing cutting time, and the two curves converge toward the end of the cutting path. Several factors may contribute to this discrepancy. First, the model assumes a constant heat flux during each tooth engagement, whereas the physical heat input is expected to vary continuously as the undeformed chip thickness decreases during down milling. Second, the finite response time of the thermocouple and the thermal contact between the thermocouple junction and the surrounding insert material may delay the experimentally observed temperature response. Third, full cutter engagement is not established instantaneously at the beginning of the cutting path. During the initial entry of the cutter into the workpiece, the effective engagement length and radial width of cut increase progressively until the nominal cutting conditions are reached. Consequently, the physical heat input during this transient entry phase is expected to be lower than that represented in the simulation, in which the nominal engagement conditions are applied from the onset of cutting. This transient entry effect was not explicitly considered in the numerical model.

Both measured and simulated temperature histories at the internal thermocouple position are smooth, without pronounced tooth-engagement-scale oscillations, while still showing the slower transient temperature rise over the cutting interval. The sensor is separated from the heat-source region by insert material. The smooth simulated response, obtained without explicitly modeling the measurement-chain dynamics, indicates that heat conduction and thermal storage within the insert attenuate rapid local temperature fluctuations before they reach this position. The finite thermocouple response may further attenuate and delay the measured response, while acquisition delay can shift its timing. Calibration therefore targets the end-of-cut temperature level rather than the amplitude or phase of local tooth-engagement fluctuations. The calibrated model is subsequently used to estimate the temperature distribution and cyclic fluctuations near the cutting edge.

Previous continuous and interrupted turning experiments supported the thermal-modeling approach through comparisons between measured and simulated temperatures at two insert locations following inverse calibration [15]. In side milling, thermocouple-based thermal reconstruction was further assessed using infrared thermography and ratio pyrometry, providing qualitative validation under dry cutting, while flood-cooling measurements relied on thermocouples [16]. These studies provide an experimental basis for the present methodology, which combines ESP32-C3-based, tool-integrated temperature measurement with inverse thermal analysis in face milling. The primary contribution is the integrated method rather than a universally applicable predictive model. The reconstructed temperatures and heat partition remain conditional on the investigated configuration and calibration, with independent validation of the present spatial temperature field remaining necessary.

**Figure 12:**
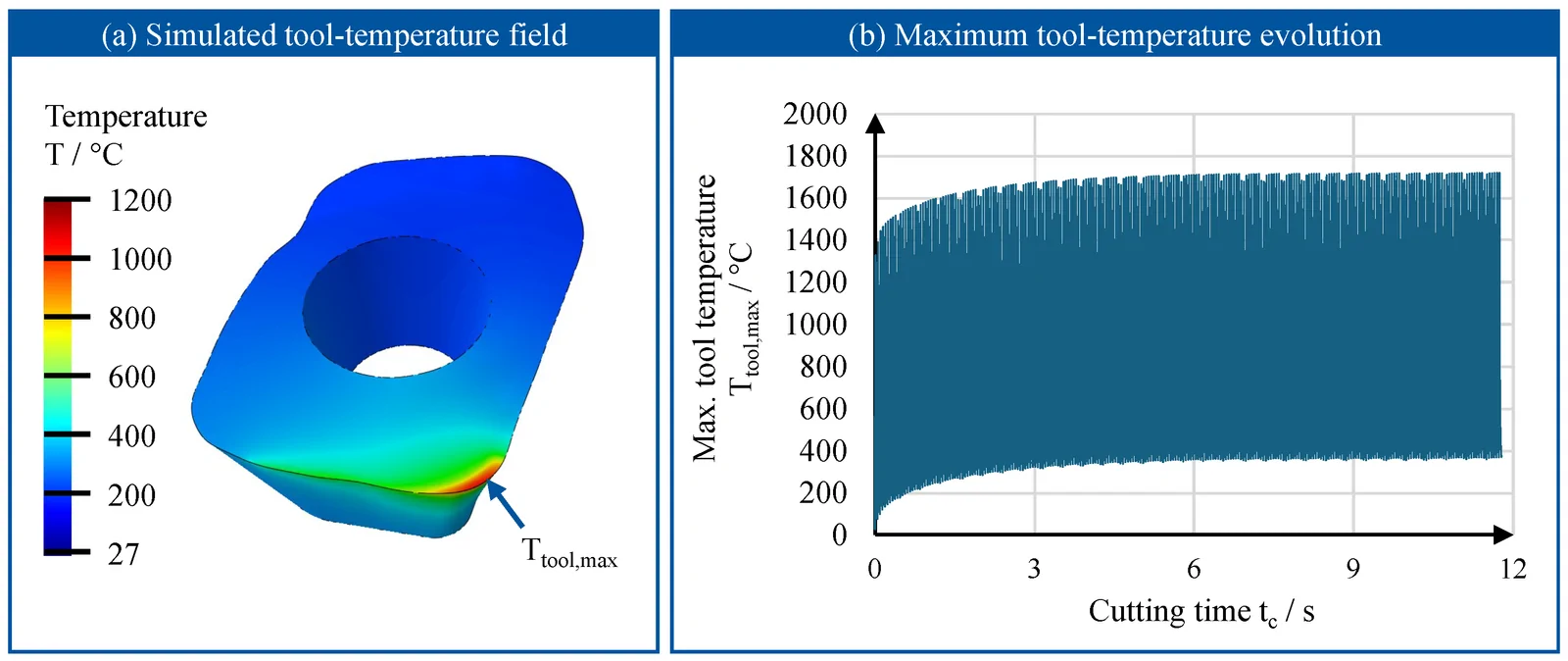
Inverse-model estimates of the temperature distribution in the cutting insert and the maximum tool-temperature evolution at $v_{c}=160\;m/\min$ and $f_{z}=300\;\mu m$.

Figure 12a illustrates the inverse-model estimate of the temperature distribution within the cutting insert for $v_{c}\;=\;160\;m/\min$ and $f_{z}\;=\;300\;\mu m$. In contrast to the temperature measured by the embedded thermocouple at a fixed position inside the insert, the model provides a conditional estimate of the local temperature field in the immediate cutting zone. Under the adopted heat-source and boundary-condition assumptions, the highest estimated temperature occurs within the heat-input region, approximately at the center of the undeformed chip cross-section. This near-edge value was not measured directly and remains dependent on the inverse calibration and model assumptions.

The simulated maximum tool temperature exhibits pronounced periodic fluctuations associated with the intermittent nature of the milling process. During each tooth engagement, heat is introduced through the tool–chip contact region, causing the local maximum temperature to rise rapidly. After the cutting edge leaves the workpiece, the heat input ceases and the temperature decreases as heat is conducted into the surrounding insert material and dissipated to the environment. This cyclic heating and cooling produces the characteristic oscillations shown in Figure 12b. At the same time, the upper and lower envelopes of the temperature response increase during the initial cutting period and gradually approach a quasi-steady level as heat accumulates within the insert.

**Figure 13:**
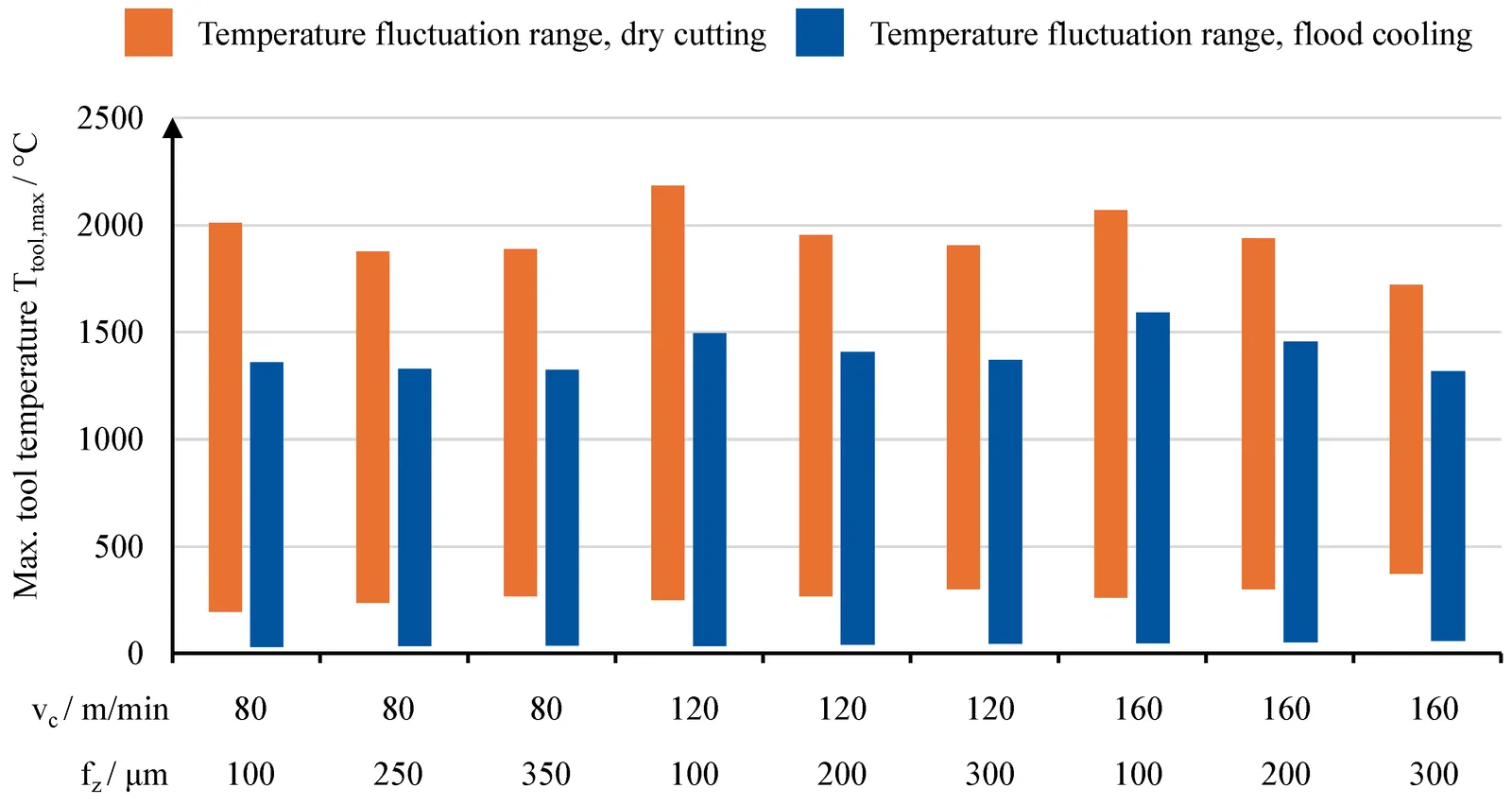
Illustrative inverse-model outputs of the maximum tool-temperature fluctuation ranges under dry cutting and flood cooling. The ranges are evaluated over the final 0.5 s of cutting using Equation (13).

The reconstructed fields complement the smooth internal thermocouple response by depicting localized cyclic heating near the cutting edge, subject to the interpretation limits in Section 3.4. Figure 13 shows the upper and lower limits of the simulated maximum-temperature histories over the final evaluation window.

The assumed heat-source geometry and temporal loading influence the reconstructed local temperature field. For a given total heat-input rate, a smaller tool–chip contact area results in a higher heat-flux density and may increase the predicted local peak temperature. Similarly, the spatial distribution of heat flux and its temporal variation during tool engagement affect the localization and evolution of thermal loading. As the influence of alternative heat-source assumptions and high-temperature material properties has not been quantified through sensitivity analysis, the predicted peak magnitudes and differences between cutting conditions are interpreted within the adopted modeling framework.

Within the calibrated simulations, the upper temperature limit generally decreases with increasing feed per tooth at a given cutting speed, whereas the lower temperature limit tends to increase slightly. Consequently, the predicted amplitude of the cyclic temperature fluctuations decreases at higher $f_{z}$. This behavior indicates that, within the adopted model, the reconstructed local peak temperature is influenced by more than cutting-force magnitude or total heat input during an individual engagement. Changes in feed per tooth affect the assumed heat-source geometry, calibrated heat-flux density, and transient heat conduction within the insert. Under dry-cutting conditions, the highest reconstructed local peak temperature occurs at $v_{c}=120$ m/min. The increase from 80 to 120 m/min is associated with greater thermal loading in the calibrated model, whereas the shorter engagement duration and lower derived tool heat-partition ratio at 160 m/min contribute to reduced heat transfer into the insert during individual engagements.

Under flood-cooling conditions, the simulations predict lower local temperatures than the corresponding dry-cutting cases when the prescribed heat input is maintained and the effective convective heat-transfer coefficient is calibrated against the measured internal temperature. Within this framework, enhanced heat removal between successive engagements reduces thermal accumulation and the predicted near-edge temperature peaks. This behavior is qualitatively consistent with the experimentally observed reduction in internal tool temperature under flood cooling. However, the magnitude of the reconstructed near-edge temperature reduction remains dependent on the adopted heat-input and thermal boundary assumptions. Published tool-flank temperature measurements [10] provide additional context for the predicted thermal behavior, although differences in measurement locations and cutting conditions limit direct quantitative comparison.

**Figure 14:**
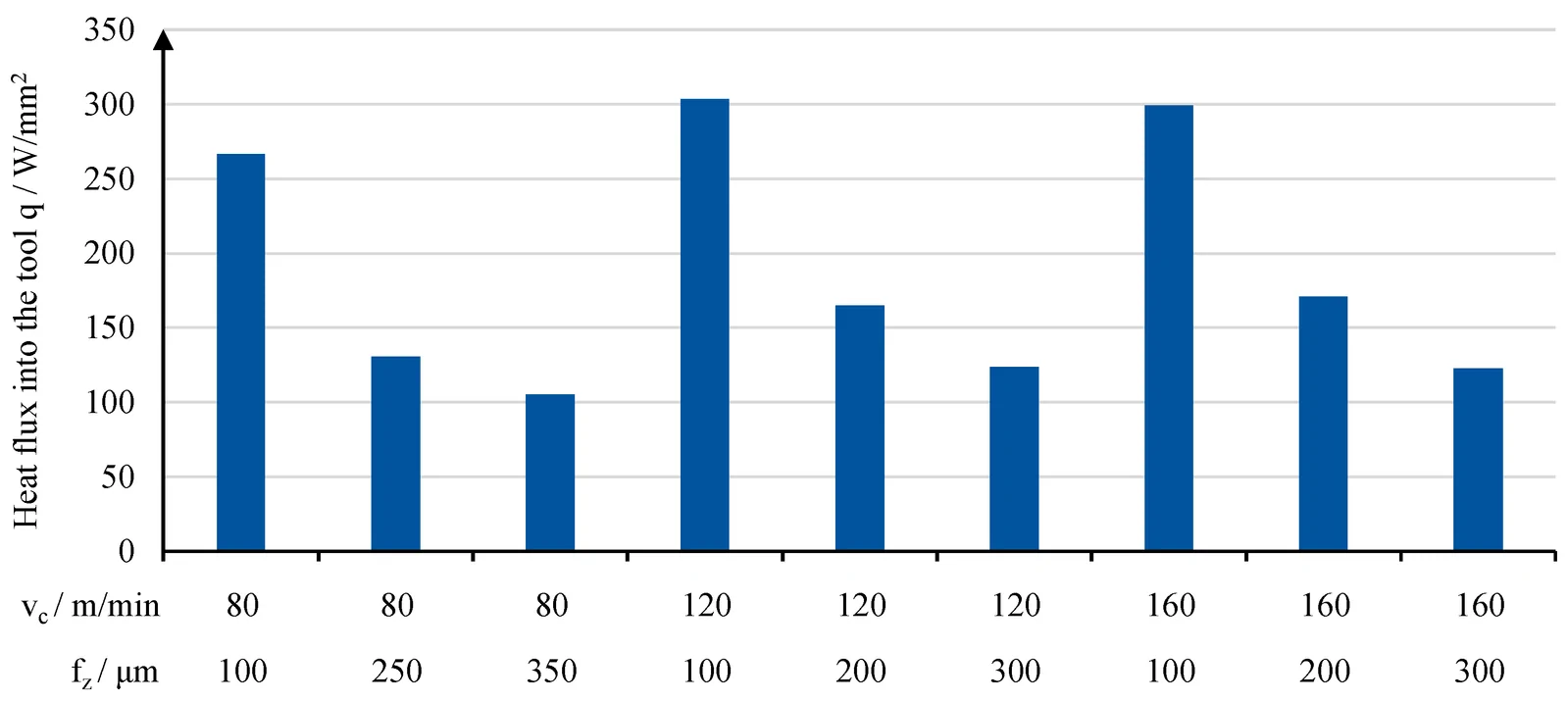
Inverse-calibrated effective heat flux into the cutting tool, in $W\;{\mathrm{mm}}^{-2}$, for different cutting parameters.

The reconstructed local temperature trends can be further interpreted using the inverse-calibrated effective heat-flux density into the cutting tool, as presented in Figure 14. For all investigated cutting speeds, the calibrated heat-flux density decreases markedly with increasing feed per tooth. At $v_{c}\;=\;80$ m/min, for example, it decreases from $q\;=\;266\;W\;{\mathrm{mm}}^{-2}$ at $f_{z}\;=\;100\;\mu m$ to $q\;=\;105\;W\;{\mathrm{mm}}^{-2}$ at $f_{z}\;=\;350\;\mu m$. Similar model-derived trends are observed at $v_{c}=120$ and 160 m/min. This decrease in effective heat-flux density should be distinguished from the total heat generated during cutting. Within the adopted heat-source representation, increasing feed per tooth enlarges the assumed tool–chip contact area over which the thermal load is distributed. Consequently, the calibrated average heat-flux density may decrease even when the total mechanical and thermal loading associated with an individual engagement increases. At the lowest feed per tooth, the calibrated heat flux rises from 266 $W\;{\mathrm{mm}}^{-2}$ at 80 m/min to 300 $W\;{\mathrm{mm}}^{-2}$ at 120 m/min, with little further change at 160 m/min. Values at 120 and 160 m/min are also similar at higher feeds. The largest flux coincides with the highest reconstructed local peak. At 160 m/min, the shorter engagement reduces modeled heat input per passage despite comparable heat flux.

**Figure 15:**
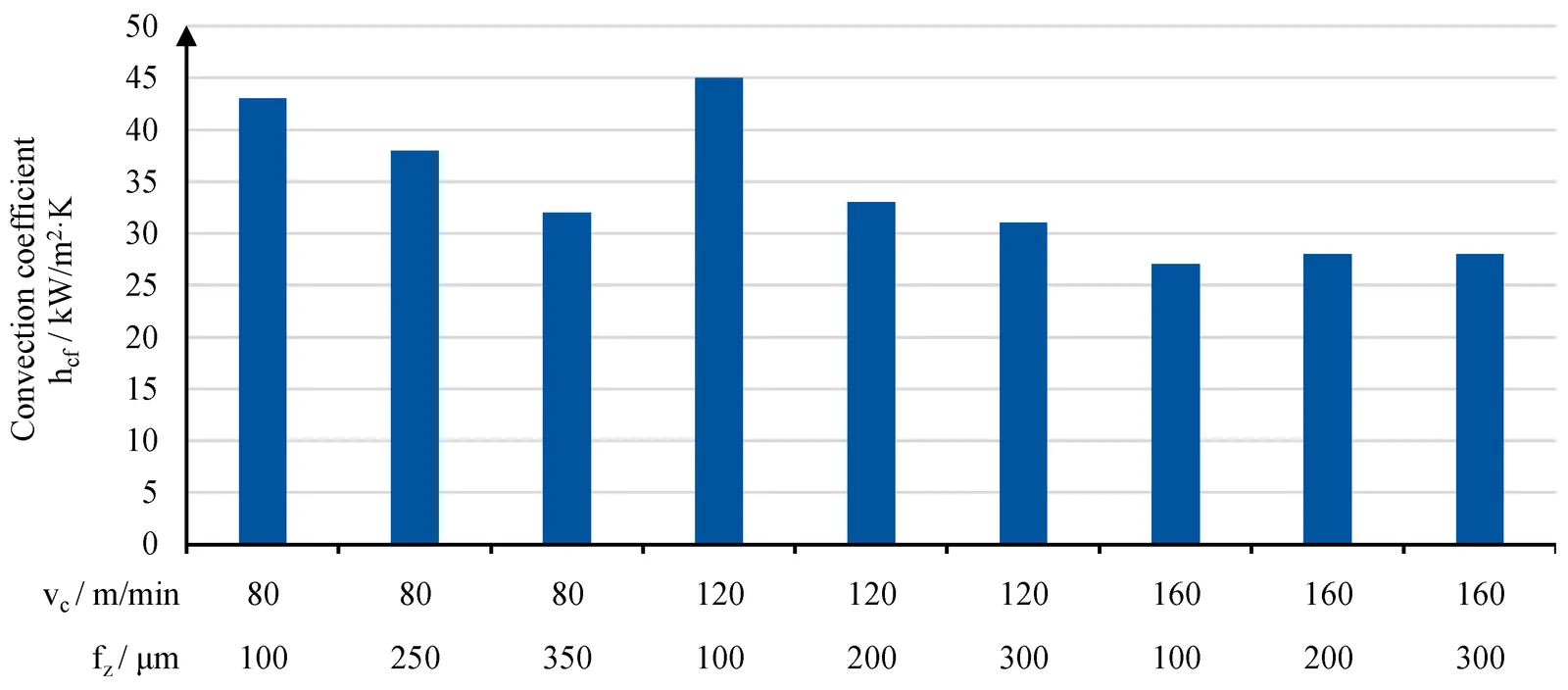
Inverse-calibrated effective convective heat-transfer coefficient, in $\mathrm{kW}\;m^{-2}\;K^{-1}$, under flood cooling for different cutting parameters.

Figure 15 presents the inverse-calibrated effective convective heat-transfer coefficients under flood cooling for the investigated cutting conditions. The fitted values range from $h_{\mathrm{cf}}$ = 27 to 45 $\mathrm{kW}\;m^{-2}\;K^{-1}$ and vary with cutting speed and feed per tooth within the investigated dataset. At $v_{c}$ = 80 m/min, the fitted coefficient decreases from $h_{\mathrm{cf}}$ = 43 to 32 $\mathrm{kW}\;m^{-2}\;K^{-1}$ as the feed per tooth increases. A similar, and more pronounced, trend occurs at $v_{c}$ = 120 m/min, where $h_{\mathrm{cf}}$ decreases from 45 to 31 $\mathrm{kW}\;m^{-2}\;K^{-1}$. One possible interpretation is that increasing undeformed chip thickness modifies coolant accessibility to the cutting zone: larger chips and higher mechanical loading may locally obstruct or redirect the cutting-fluid flow. This interpretation is not an independent measurement of local fluid mechanics.

At $v_{c}$ = 160 m/min, the fitted effective heat-transfer coefficients are lower overall and remain nearly constant at $h_{\mathrm{cf}}$ = 27 to 28 $\mathrm{kW}\;m^{-2}\;K^{-1}$ over the investigated range of feed per tooth. Among these fitted values, the variation with feed per tooth is small. The lower calibrated coefficients at $v_{c}$ = 160 m/min may be associated with changes in coolant accessibility and interaction time caused by the higher rotational speed, but this proposed mechanism requires independent fluid-flow or local heat-transfer measurements. It should be emphasized that $h_{\mathrm{cf}}$ represents an effective parameter calibrated from the measured tool-temperature response rather than a directly measured local fluid-mechanical heat-transfer coefficient. Within the model, the effective convective heat-transfer coefficient represents the combined influence of cutting-fluid impingement, wetting, fluid redistribution around the cutting zone, and intermittent insert exposure. The model response therefore reflects the interaction of the prescribed heat input, loading duration, and fitted effective convection.

**Figure 16:**
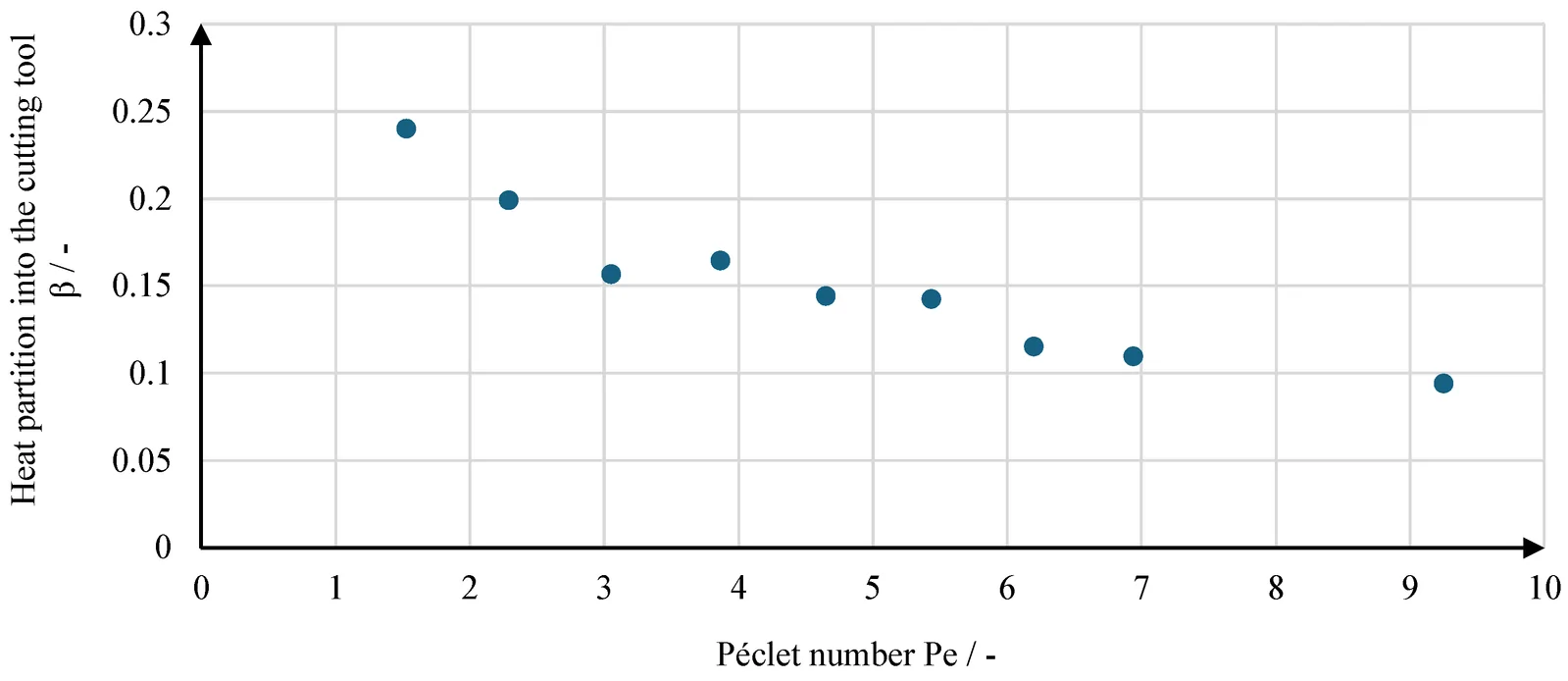
Model-derived heat partition into the cutting tool as a function of the Péclet number.

The model-derived relationship between cutting parameters and heat partitioning was examined using the Péclet number Pe. Figure 16 shows the heat-partition ratio into the cutting tool, β, derived from the inverse-calibrated model under the stated assumptions, as a function of the Péclet number for all investigated cutting conditions. To account for the combined effects of cutting speed and undeformed chip thickness on transient heat transfer, the Péclet number was calculated as

(19) $$\begin{array}{c} \mathrm{Pe}=\frac{v_{c}h}{\alpha } \end{array}$$

where $v_{c}$ is the cutting speed, h is the equivalent undeformed chip thickness, and α is the thermal diffusivity of the cutting-tool material.

For the present Péclet-number calculation, *h* was determined geometrically by dividing the heat-source area $A_{q}$ by the length $L_{e}$ of the contact region along the cutting edge:

(20) $$h=\frac{A_{q}}{L_{e}}.$$

Table 7 lists the heat-source areas and the resulting equivalent thicknesses for all nine cutting conditions. The along-edge contact length was 6.68 mm in every case. Thus, *h* represents an area-averaged characteristic width of the heat-source region and is not set equal to the feed per tooth. The length $L_{e}$ runs along the cutting edge and is distinct from the tool–chip contact length in the chip-flow direction discussed in Section 3.1.

With the along-edge length held constant, increasing feed per tooth increases the heat-source area and hence *h*. For example, at 80 m/min, increasing $f_{z}$ from 100 to 350 μm increases $A_{q}$ from 0.30 to 1.07 ${\mathrm{mm}}^{2}$ and *h* from approximately 0.04491 to 0.16018 mm. For equal feed per tooth, the listed geometry is unchanged across cutting speeds. Both the speed and this geometrically defined thickness therefore contribute to the Péclet number, with $v_{c}$ and *h* converted to $m\;s^{-1}$ and metres, respectively, when using thermal diffusivity in $m^{2}\;s^{-1}$.

The thermal diffusivity was calculated from the thermal conductivity k, density ρ, and specific heat capacity $c_{p}$ according to

(21) $$\begin{array}{c} \alpha =\frac{k}{\rho c_{p}} \end{array}$$

Using the room-temperature properties in Table 4, the thermal diffusivity was calculated as $\alpha =3.93\times 10^{-5}\;m^{2}\;s^{-1}$ [15].

The nine model-derived heat-partition values exhibit a descriptive decrease with increasing Péclet number. At the lowest investigated value, Pe=1.52, the inverse-calibrated model assigns a heat-partition ratio of 24% to the tool. This derived fraction decreases to 9.4% at Pe=9.24, corresponding to a reduction of more than 60% across the investigated conditions. Despite a local deviation around Pe=3.86, the present inverse results show a descriptive negative association between Pe and β. This relationship has not been independently validated.

The heat-partition ratio is calculated from the inverse-calibrated heat input and the corresponding mechanical cutting energy according to Equation (9). Its quantitative value therefore depends on the assumed heat-source geometry, thermal boundary conditions, and mechanical-to-thermal energy conversion factor, as well as the measured cutting power. Within these assumptions, the calculated values provide a consistent basis for examining the relationship between heat partitioning and the Péclet number across the investigated cutting conditions. Although the absolute heat-partition values remain model-dependent, the observed trend provides insight into the influence of cutting conditions on the distribution of thermal energy within the adopted modeling framework.

For comparison, Liu et al. [27] reported inverse-model tool heat fractions of approximately 9.5–15% for orthogonal cutting of AISI 1045 steel at $v_{c}$ = 80–120 m/min and equivalent chip thicknesses of 0.20–0.30 mm. The present range of 9.4–24% encompasses the reported interval and extends to a higher upper bound. Differences in cutting kinematics, workpiece and tool materials, geometry, and inverse boundary conditions preclude direct validation. Nevertheless, the descriptive tendency toward a lower tool heat fraction with increasing Péclet number is consistent with earlier heat-partition modeling [27,31].

One possible interpretation of this model-derived trend concerns the relative time scales of material transport and heat conduction in the cutting zone. At lower Péclet numbers, a longer interaction time may allow a larger fraction of the generated heat to conduct through the tool–chip interface into the cutting insert. At higher Pe, material transport may carry more heat away with the chip before it can conduct into the tool. However, the decrease in the inferred tool heat fraction does not by itself establish an increase in the chip heat fraction, because the chip and workpiece contributions were not separately determined. Increased heat removal by the chip is therefore a possible mechanism requiring independent assessment, and this interpretation remains conditional on the inverse-model assumptions. Within this model, the decreasing tool heat fraction with increasing Péclet number complements the engagement-time explanation of the non-monotonic local temperature response.

Overall, the inverse-model results suggest that the reconstructed thermal loading cannot be described by cutting speed or feed per tooth alone. By combining cutting speed, characteristic chip thickness, and tool-material thermal diffusivity, the Péclet number may provide a descriptive parameter within the investigated dataset. The model-derived decrease in β with increasing Pe offers an internally consistent, but model-dependent, interpretation of the reconstructed insert-temperature behavior.

## 5. Conclusions and Outlook

This study investigated tool-temperature evolution and heat partitioning during face milling under dry-cutting and flood-cooling conditions using an integrated ESP32-C3-based measurement system and inverse finite element thermal analysis. Electrical verification of the signal-conditioning and acquisition chain at ten reference temperature setpoints yielded a pooled RMSE of 1.523 °C. Comparison with the commercial MANNER telemetry system in separate cutting trials across nine dry-cutting conditions resulted in RMSDs of approximately 1.8–3.4 °C, indicating good agreement between the recorded temperature histories under matched cutting parameters. These results provide complementary assessments of the electronic acquisition performance and experimental measurement consistency. The compact, sealed configuration of the developed system additionally enabled reliable temperature acquisition under flood-cooling conditions, demonstrating its practical potential for tool-integrated thermal monitoring in machining applications.

The experimental results showed that feed per tooth exerted the most pronounced influence on the measured internal tool temperature under dry-cutting conditions and on cutting forces under both cooling conditions. Under dry cutting, internal tool temperatures ranged from approximately 175 to 235 °C and generally increased with increasing feed per tooth. Cutting speed exhibited a comparatively smaller influence, reflecting the combined effects of thermal loading, engagement duration, and heat partitioning. Flood cooling reduced the measured internal temperatures to generally below 70 °C and substantially decreased their sensitivity to the investigated cutting parameters. At $v_{c}=160$ m/min, temperatures remained close to 50 °C across the investigated feed range. In contrast, the cutting-force distributions under dry and flood cooling largely overlapped, suggesting that the applied cooling conditions had a more pronounced influence on the thermal response than on the measured mechanical loading.

The three-dimensional finite element model was calibrated individually for each cutting condition using the measured end-of-cut temperature at the thermocouple position. The simulated temperature histories reproduced the general heating behavior, although a faster initial temperature increase was observed in the simulation for the reference condition. Both measured and simulated temperatures at the internal sensor position exhibited smooth temporal evolution, whereas the reconstructed temperature field showed more pronounced cyclic thermal loading near the cutting edge. The model predicted local peak temperatures of approximately 1700–2200 °C under dry cutting and 1300–1600 °C under flood cooling. These values represent model-dependent estimates of localized thermal loading rather than directly measured temperatures.

The inverse thermal analysis further provided estimates of effective heat input, cooling intensity, and heat partitioning. The calibrated effective heat flux decreased with increasing feed per tooth, consistent with the larger assumed tool–chip contact area at greater undeformed chip thickness. Under flood cooling, the effective convective heat-transfer coefficient ranged from approximately 27 to 45 $\mathrm{kW}\cdot m^{-2}\cdot K^{-1}$ and remained nearly constant at approximately 27–28 $\mathrm{kW}\cdot m^{-2}\cdot K^{-1}$ for $v_{c}\;=\;160$ m/min. The model-derived fraction of heat transferred into the tool decreased from 24% at Pe=1.52 to 9.4% at Pe=9.24, indicating a decreasing trend with increasing Péclet number within the investigated process window. This relationship provides a preliminary description of heat partitioning based on the calibrated thermal model.

The reconstructed near-edge temperatures, calibrated effective heat fluxes and convection coefficients, and derived heat-partition ratios are interpreted within the prescribed geometry, thermal boundary conditions, and material-property assumptions, including the constant extension beyond the tabulated temperature ranges. The inverse-calibrated model provides a consistent framework for investigating local thermal behavior, while independent validation and systematic sensitivity analyses would further strengthen the quantitative assessment of the reconstructed temperature fields and identified parameters. In particular, the absolute peak temperatures and their differences between cutting conditions should be interpreted as model-dependent estimates when considering applications such as thermal damage assessment or tool-life prediction. Furthermore, the limited number of experimental repetitions and the use of unworn inserts constrain the generalizability of the findings to other machining conditions and tool-wear states.

Future investigations should focus on independent validation of the reconstructed temperature field and extension of the method to different tool geometries, workpiece materials, wear states, and cooling strategies. Additional temperature measurements at multiple locations or depths, together with complementary optical measurements under dry cutting conditions, could provide independent references for assessing spatial temperature predictions. Systematic sensitivity analyses should examine the influence of contact length, spatially and temporally varying heat flux, flank-face heating, tool-holder transients, and coolant boundary conditions. High-temperature material-property characterization would further improve the assessment of predicted cutting-zone temperatures. These developments could extend the application of the integrated measurement and numerical analysis approach to thermal process monitoring, tool-wear investigations, and thermally informed machining optimization.

## Figures and Tables

**Table 1:** Publicly available reference prices for the principal electronic components of the developed measurement system (EUR per unit, excluding VAT and shipping; accessed on 24 October 2026).

Component	EUR	Source
XIAO ESP32C3 (113991054)	4.22	Farnell [23]
AD8495 breakout (Adafruit 1778)	15.70	Opencircuit [24]
MicroSD breakout (Adafruit 254, DEBO MICROSD)	8.49	Reichelt [25]; EUR 10.10/1.19
Subtotal: three core electronic modules	28.41	One unit of each module

**Table 2:** Measured chemical composition of the quenched-and-tempered (QT) 42CrMo4 workpiece material.

Element	wt.%	Element	wt.%	Element	wt.%	Element	wt.%
C	0.392	Si	0.238	Mn	0.805	P	0.0196
S	0.0087	Cr	1.15	Mo	0.192	Ni	0.132
Al	0.0179	Co	0.0049	Cu	0.194	Nb	0.0021
Ti	0.0011	V	0.0055				

**Table 3:** Cutting-speed and feed-per-tooth combinations used in the milling experiments.

Cutting speed $v_{c}$/m/min	80	120	160
Feed per tooth $f_{z}$/μm	100, 250, 350	100, 200, 300	100, 200, 300

**Table 4:** Temperature-dependent thermal properties of the cutting-insert material.

Thermal Conductivity		Specific Heat Capacity	
k	T	$c_{p}$	T
$W\;m^{-1}\;K^{-1}$	°C	$J\;{\mathrm{kg}}^{-1}\;K^{-1}$	°C
115.17	25	198	25
106.25	100	215	100
96.17	200	229	200
88.44	300	238	300
81.78	400	243	400
75.92	500	246	500
		250	1500

**Table 5:** Electrical verification against the Fluke 714 setpoints (all values are in °C).

Setpoint	Bias	SD	RMSE	Setpoint	Bias	SD	RMSE
25.2	−0.359	0.057	0.364	150.2	+2.192	0.074	2.193
50.2	+0.144	0.137	0.198	175.1	+0.383	0.325	0.503
75.0	+2.161	0.275	2.179	200.2	−0.068	0.340	0.346
100.0	+2.436	0.108	2.438	225.2	+0.536	0.317	0.623
125.2	+2.449	0.099	2.451	250.2	+0.965	0.241	0.995

**Table 6:** Agreement between temperature histories obtained from the ESP32-based and MANNER systems in separate dry-cutting trials.

$v_{c}$ (m/min)	80	80	80	120	120	120	160	160	160
$f_{z}$ (μm)	100	250	350	100	200	300	100	200	300
RMSD (°C)	3.1	3.4	2.3	2.9	2.7	2.2	2.6	2.0	1.8
${\bar{\delta }}_{j}$ (°C)	+2.3	+1.7	+1.9	+1.8	+1.4	−0.4	−0.4	−0.3	+0.7

**Table 7:** Heat-source area, along-edge contact length, and equivalent thickness used to define the Péclet number.

$v_{c}$ (m/min)	80	80	80	120	120	120	160	160	160
$f_{z}$ (μm)	100	250	350	100	200	300	100	200	300
$A_{q}$ (${\mathrm{mm}}^{2}$)	0.30	0.76	1.07	0.30	0.61	0.91	0.30	0.61	0.91
$L_{e}$ (mm)	6.68	6.68	6.68	6.68	6.68	6.68	6.68	6.68	6.68
*h* (mm)	0.04491	0.11377	0.16018	0.04491	0.09132	0.13623	0.04491	0.09132	0.13623

## Data Availability

The data supporting the findings of this study are included in the article. Further inquiries can be directed to the corresponding author.

## Abbreviations

The following abbreviations are used in this manuscript:

ADC	Analog-to-digital converter
AISI	American Iron and Steel Institute
CT	Computed Tomography
CVD	Chemical Vapor Deposition
ESP	ESP32-C3-based measurement system
FEM	Finite Element Method
FM	Frequency Modulation
HSK	Hohlschaftkegel (Hollow-Shank Taper)
ISO	International Organization for Standardization
NI	National Instruments
PLA	Polylactic Acid
PCM	Pulse-Code Modulation
QT	Quenched and Tempered
SoC	System-on-Chip
STEP	Standard for the Exchange of Product Model Data
